# Origin and Fates of *TERT* Gene Copies in Polyploid Plants

**DOI:** 10.3390/ijms22041783

**Published:** 2021-02-11

**Authors:** Petr Fajkus, Vratislav Peška, Jiří Fajkus, Eva Sýkorová

**Affiliations:** 1 Institute of Biophysics of the Czech Academy of Sciences, Královopolská 135, CZ-61265 Brno, Czech Republic; fajkuspe@ibp.cz (P.F.); vpeska@ibp.cz (V.P.); 2 Laboratory of Functional Genomics and Proteomics, NCBR, Faculty of Science, Masaryk University, Kotlářská 2, CZ-61137 Brno, Czech Republic; 3 Mendel Centre for Plant Genomics and Proteomics, CEITEC, Masaryk University, Kamenice 5, CZ-62500 Brno, Czech Republic

**Keywords:** polyploidy, *Nicotiana*, telomerase, gene evolution, synteny

## Abstract

The gene coding for the telomerase reverse transcriptase (*TERT*) is essential for the maintenance of telomeres. Previously we described the presence of three *TERT* paralogs in the allotetraploid plant *Nicotiana tabacum*, while a single *TERT* copy was identified in the paleopolyploid model plant *Arabidopsis thaliana*. Here we examine the presence, origin and functional status of *TERT* variants in allotetraploid *Nicotiana* species of diverse evolutionary ages and their parental genome donors, as well as in other diploid and polyploid plant species. A combination of experimental and in silico bottom-up analyses of *TERT* gene copies in *Nicotiana* polyploids revealed various patterns of retention or loss of parental *TERT* variants and divergence in their functions. RT–qPCR results confirmed the expression of all the identified *TERT* variants. In representative plant and green algal genomes, our synteny analyses show that their *TERT* genes were located in a conserved locus that became advantageous after the divergence of eudicots, and the gene was later translocated in several plant groups. In various diploid and polyploid species, translocation of *TERT* became fixed in target loci that show ancient synapomorphy.

## 1. Introduction

Flowering plants (angiosperms) are important for the existence of many terrestrial organisms, including humans, and a long history of plant breeding has taught us that polyploidization can be advantageous in terms of quantitative traits of crops. Gains and losses of paralogs, their neofunctionalization and sub-functionalization, have all been associated with the generation of duplicate gene copies, e.g., by whole-genome duplications (WGDs) and further rounds of genome duplication/reduction, resulting in genetic diversity upon which the fittest combinations thrived in a competitive environment [1,2,3,4]. An ancient WGD has been reconstructed at the base of seed plants, another at the base of angiosperms [5,6,7] and numerous additional, subsequent WGD events were associated with the divergence of many angiosperm lineages [3]. Polyploidy is usually associated with many genetic and epigenetic changes, including chromosomal rearrangements, expansions of transposable elements and changes in gene expression [8,9]. At the gene level, polyploids can tolerate the presence of paralogs or eliminate a copy of the spare gene. Thus, evolutionary forces result in an equilibrium defined by gene dosage [10]. Studies of model plants have mostly focused on genes important for crop production; however, genes that are critical for genome stability are extremely important for understanding repeated polyploidization events during natural selection, and these remain underexplored.

**Figure 1:**
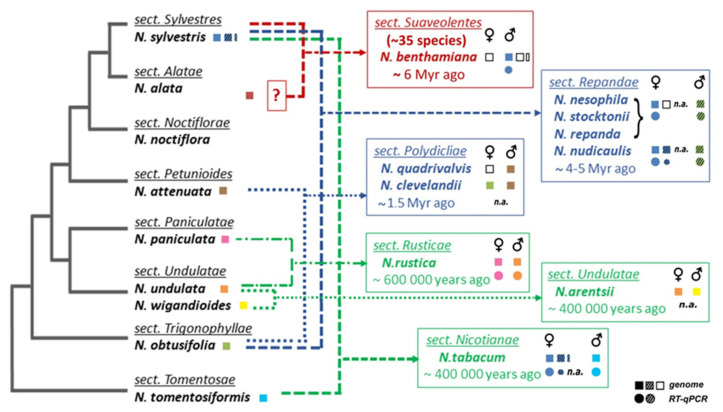
Overview of experimental results and illustration of phylogenetic relationships of *Nicotiana* species used in this study. Phylogeny and the proposed origin of polyploids were adapted from [31,32]. An uncertain parental genome donor for section Suaveolentes is indicated by a question mark. Summary of experimental and in silico results (squares, analyses of genomic DNA; circles, expression of *TERT* variants investigated by RT–qPCR) is shown in boxes of Nicotiana sections, the origin of *TERT* variant in polyploids is depicted by color of respective parental diploids, and variants that were not identified are depicted with open squares. *Nicotiana* accessions used in the experimental analyses are listed in Table S1, genomic assemblies and genomic/transcriptomic SRA data used for in silico analyses are listed in Material and Methods. For the purposes of this paper, we refer to a *TERT* copy that does not code for a catalytically active protein as a putative pseudogene (dashed symbols) in contrast to a functional *TERT* gene copy (open symbols), n.a. not analyzed.

Telomerase reverse transcriptase (TERT) is involved in the maintenance of telomeres, nucleoprotein structures that are essential for genome stability [11,12,13]. Telomerase adds telomere repeats to the ends of eukaryotic chromosomes, thereby elongating telomeres and compensating for their shortening due to incomplete end-replication. When telomerase is not active, telomeres become shortened, and their function in the protection of chromosomes is disrupted. The extreme evolutionary success of telomerase-based mechanisms of telomere maintenance is illustrated by current findings in plants (reviewed in [14]). Even among apparent exceptions in telomere sequences, in plant genera *Allium* (Asparagales) and *Cestrum* (Solanales) [15,16,17,18], recent research has revealed that novel, unusual telomere DNA sequences are synthesized by telomerase [16,18,19] and not by alternative mechanisms as had been suggested previously (reviewed in [20]). Moreover, we recently demonstrated that changes in the template region of the telomerase RNA subunit directed the observed evolutionary transitions in telomere DNA sequences [14,21,22]. In contrast to the RNA subunit, the protein subunit TERT is evolutionary well conserved and possesses a central reverse transcriptase domain essential for its catalytic function [23,24]. Plant TERTs are structurally similar to human, ciliate or yeast TERTs with a telomerase-specific T motif [25,26,27,28,29]. The gene encoding TERT is usually expressed at low mRNA levels even in telomerase-positive tissues and is maintained as a single copy gene in most eukaryotic genomes. However, the natural allotetraploid *Nicotiana tabacum* possesses three sequence variants of the *TERT* gene [30]. Various allopolyploidization events among closely and distantly related diploid parental species (Figure 1) in *Nicotiana* make the genus an ideal experimental model system to study the long-term evolution of *TERT* following natural gene duplication. The increasing number of publicly available assembled plant genomes enables the exploration of *TERT* genomic loci, gene copy numbers and gene synteny in diverse plant species for comparisons with the data from *Nicotiana* polyploids and the diploid species most closely related to their progenitors (hereafter called progenitor diploids). The *Nicotiana* genus [31,32,33,34,35] comprises relatively young polyploids (i) *N. tabacum* (section Nicotianae), *N. rustica* (sect. Rusticae), *N. arentsii* (sect. Undulatae) that formed approx. 0.4–0.6 million years ago, (ii) *N. clevelandii* and *N. quadrivalvis* (ca. 1.5 million years ago, sect. Polydicliae), (iii) four species from the 4–5 million years old section Repandae (*N. nudicaulis*, *N. repanda*, *N. nesophila* and *N. stocktonii*), and (iv) ~35 species including the model *N. benthamiana* from the oldest section Suaveolentes formed about 6 million years ago [31]. Among these species, members of sections Suaveolentes and Repandae are of interest because, with *N. tabacum*, they share an ancient genome donor, *N. sylvestris*, and these speciation events happened at different times. In *N. tabacum*, two *TERT* variants originated from the maternal *N. sylvestris* genome (*TERT*_Cs, *TERT*_D) and one from the *N. tomentosiformis* paternal genome (*TERT*_Ct). Variants *TERT*_Cs and *TERT*_Ct code for a full-length functional protein, while the *TERT*_D variant is truncated and contains several indels resulting in premature stop codons, suggesting that it is a pseudogene [30]. All three variants are nevertheless transcribed and show distinct, tissue-dependent levels of mRNA transcripts, indicating a sub-functionalization of *TERT* variants [30,36].

Based on previously described *TERT* variants in *N. tabacum*, we explored the fate of *TERT* paralogs in other *Nicotiana* polyploids to determine whether both parental *TERT* genes are conserved in allotetraploid genomes, whether they are transcribed, present in syntenic, collinear arrays with their progenitor diploids, and whether any relationship exists between telomere lengths in polyploids and their progenitor diploids. Of particular interest in this study was to clarify the origin of the presumed pseudogene variant *TERT_D* in *N. sylvestris*, a diploid genome donor of *N. tabacum*, as well as of even older species from sections Repandae and Suaveolentes. In addition, we investigated in silico whether diploid and polyploid plants outside of the family Solanaceae sustained *TERT* paralogs/pseudogenes in their genomes, and we explored syntenic relationships of genes adjacent to *TERT* to interpret the evolutionary success of *TERT* copies after translocation.

## 2. Results

### 2.1. Number of TERT Variants in Nicotiana Polyploids as a Case Study

**Figure 2:**
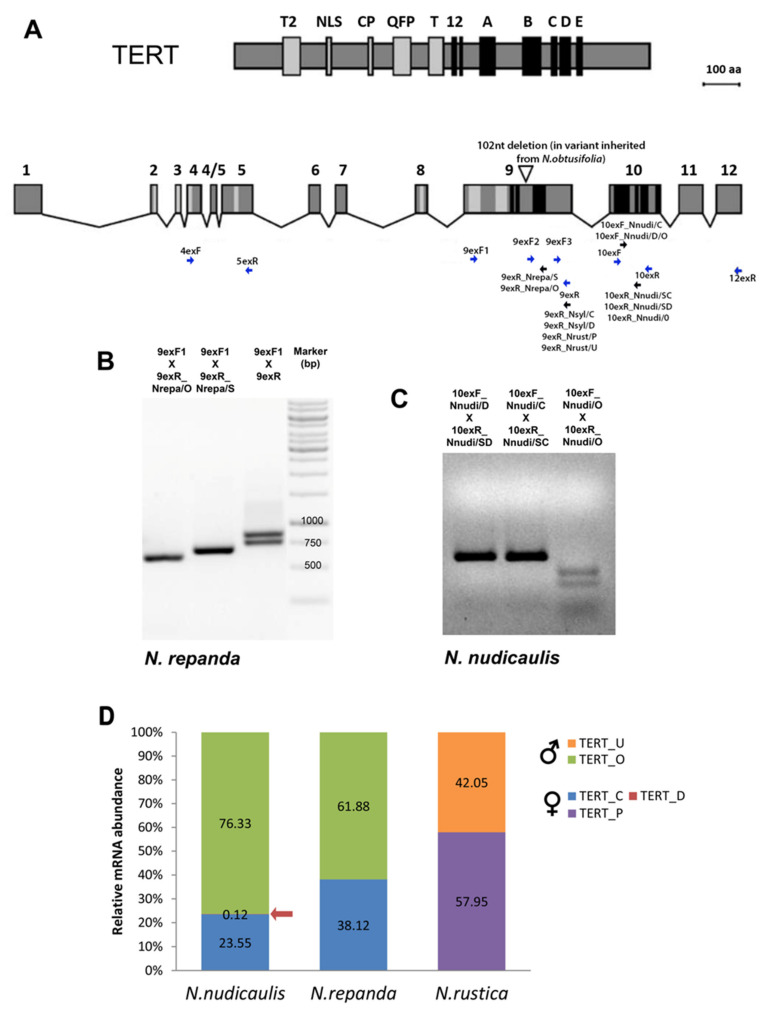
Experimental identification of TERT variants and analysis of gene expression in *Nicotiana* polyploids. (**A**) Conserved telomerase specific motifs (T2, NLS, CP, QFP, T) and reverse transcriptase motifs (1, 2, A–E) are highlighted in protein and mRNA of *Nicotiana* TERT (modified from [30]). Positions of primers used for screening experiments (blue arrows) and *TERT*-variant-specific primers (black arrows) are indicated at corresponding *TERT* mRNA regions (primers are listed in Table S2). The triangle within exon 9 shows the position of a 102 nt long deletion that was identified in *N. repanda*, *N. nesophila* and *N. stocktonii* and represents a specific *TERT*-variant of *N. obtusifolia* origin. (**B**,**C**) Validation of primer specificity for *TERT* variants in *N. repanda* (**B**) and *N. nudicaulis* (**C**). PCR products amplified with primers 9exF1 and 9exR1 show two bands corresponding to *TERT*_O and *TERT*_Cs variants that differ by a 102 bp long deletion. Specific amplification of *TERT*_O and *TERT*_Cs variants was demonstrated using the 9exF1 primer in combination with variant-specific reverse primers 9exR_Nrepa/O and 9exR_Nrepa/S, respectively. (**C**) For validation of qPCR primers and to distinguish three *TERT* variants in *N. nudicaulis*, the PCR products amplified with indicated qPCR primer combinations were digested with *Mse*I. A specific cut of the *TERT*_O variant that possesses the restriction site for *Mse*I within the amplified region confirmed the specificity of amplified *TERT*-variants. (**D**) Relative mRNA levels of specific *TERT* variants were determined by RT–qPCR in *N. nudicaulis*, *N. repanda* and *N. rustica*. Relative mRNA abundance of particular parental *TERT* variants (in %) was calculated by the delta Ct method [37]. Ct values were normalized using the reaction efficiency calculated from a standard curve analysis (Table S3).

At the beginning of this project, there was limited genomic sequence data available for the majority of *Nicotiana* allopolyploids and their parents. To characterize experimentally the number, identity and origin of *TERT* copies in genomes of polyploid *Nicotiana* species and representatives of their diploid progenitors, we employed several primer combinations derived from conserved *TERT* regions of the evolutionarily distant relatives *N. sylvestris* and *N. tomentosiformis* (Figure 1), designed originally for amplification of *N. tabacum TERT* variants [30,36]. These PCR primers (Figure 2A, Table S2) amplify *TERT* regions nonspecifically, i.e., all variants are produced in a single PCR. Sequencing of PCR products then identifies single nucleotide polymorphisms (SNPs) and/or indels evidencing the presence of multiple *TERT* variants. Primer positions were with respect to *Nicotiana TERT* gene structure with 13 exons (Figure 2A), which differed from the prevalent 12-exon structure of plant *TERT*s [23]. As expected, a successful amplification was achieved mostly using primers derived from the more conserved sequences at the 3′ end of *TERT* genes (Table S3). As the first screening experiment, we applied this approach to six diploid *Nicotiana* species investigated as representatives of parental genome donors, including *N. sylvestris* as a control, and to nine polyploid *Nicotiana* species (Figure 1). Among parental diploids, we detected one *TERT* variant in *N. alata*, *N. attenuata*, *N. undulata*, *N. wigandoides*, *N. paniculata* and *N. obtusifolia* (Supplementary A1), and two *TERT* variants (*TERT*_C and *TERT*_D) in *N. sylvestris* [30]. In the case of *N. attenuata* and *N. obtusifolia*, species representing parents of polyploid sections Polydicliae and Repandae, we further confirmed our results by in silico analysis using genome assemblies (GenBank accessions: GCA_001879085.1 and GCA_002018475.1, respectively). To complete the set of representative parental species, we assembled available transcriptomic SRA data of *N. noctiflora* (GenBank accession: SRR2106514) and identified one *TERT* variant. In conclusion, our results show the presence of more than one *TERT* variant in diploid *N. sylvestris* [30], an exception among parental species of *Nicotiana* polyploids.

The same experimental approach applied to representative *Nicotiana* polyploids detected variant-specific SNPs and/or indels, demonstrating the presence of two *TERT* variants in 5 of 9 polyploid species investigated (*N. arentsii*, *N. rustica*, *N. repanda*, *N. nesophila*, *N. stocktonii*) and three variants were identified in *N. nudicaulis* (summarized in Figure 1, Table 1, see below for details). While PCR products obtained from *N. clevelandii, N. quadrivalvis* and *N. benthamiana* genomic DNA revealed the presence of a single copy of the *TERT* gene, our search for *TERT* variants in raw transcriptomic data from *N. clevelandii* showed the occurrence of two gene variants. To avoid possible errors in comparison of experimental and in silico data that could be caused, e.g., by possible incorrect mapping of *TERT* reads to the raw genome/transcriptome data, assembly version or allele sequence, we analyzed in detail individual SNPs in sequences from each polyploid species and its progenitor diploids (see Supplemental Text S1, Figure S1, Table S4). Results deduced from sequence similarity (in %, Table 1) and individual SNPs (Table S4) were in agreement in all cases analyzed.

### 2.2. Origin of TERT Genes in Polyploids with the Ancestral N. sylvestris Donor Genome

An *N. sylvestris* progenitor is considered to be a progenitor diploid of the allopolyploid sections Suaveolentes, Repandae and Nicotianae (Figure 1). The evolutionary history of *TERT* associated with allopolyploidy is inferred for each of these sections.

#### 2.2.1. Suaveolentes

In the section Suaveolentes, we used the model plant *N. benthamiana* as a representative species of the section, and *N. alata* and *N. noctiflora* as recent relatives of the putative maternal lineage originating from sections Alatae or Noctiflorae, respectively (Figure 1, [31,32,38]). We detected a single copy of the *N. benthamiana TERT* experimentally, and this result was confirmed in silico (Table 2) using (i) an *N. benthamiana* genome assembly based on deep sequencing (N. benthamiana Genome v1.0.1) and (ii) analysis of raw genomic NGS reads [39] by BLAST followed by read-mapping back to the query. Comparison of corresponding regions of *N. benthamiana TERT* and representative parental *TERT* sequences (Table 1, Table S4) revealed that the *N. benthamiana TERT* sequence (accession number NbS00010427g0116.1) was more similar to *N. sylvestris TERT*_C variant than to the *TERT* sequence cloned from *N. alata* (GenBank accession MG242421) or deduced from *N. noctiflora* SRA data (Supplementary A1). Thus, we conclude an *N. sylvestris* origin of *N. benthamiana TERT* and a loss of the second parental *TERT* copy during the evolution of *N. benthamiana*.

#### 2.2.2. Repandae

**Figure 3:**
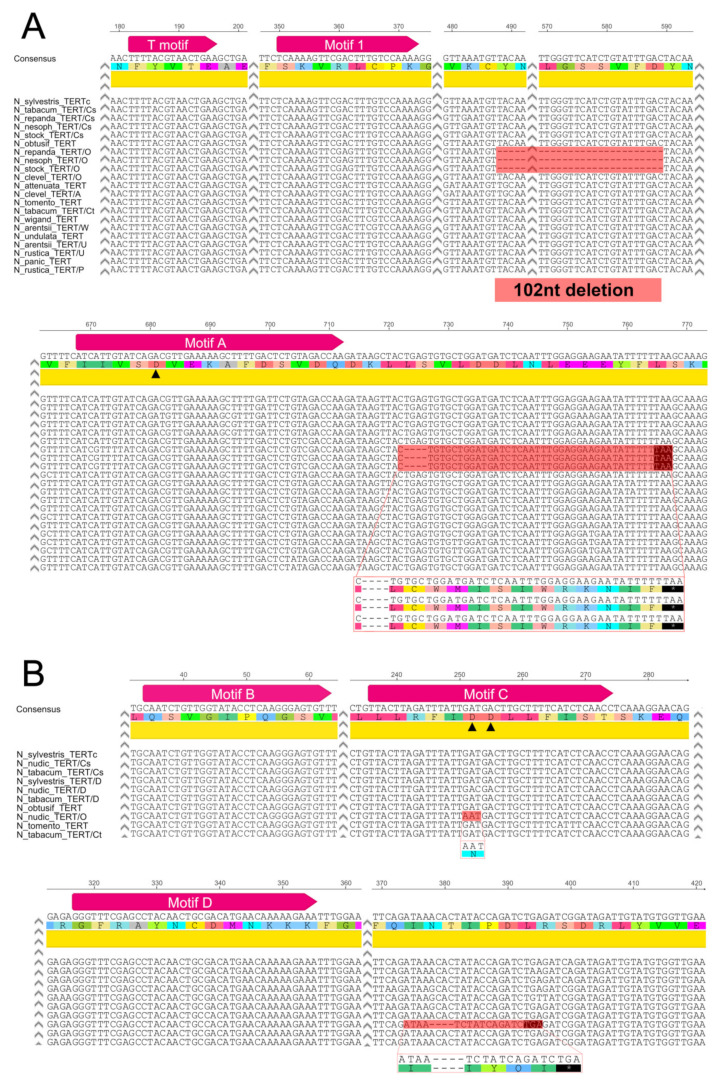
Comparison of *TERT* variants. (**A**) Alignment of representative *TERT* variants from *Nicotiana* polyploids identified here, and previously in *N. tabacum* [30], clearly illustrates the occurrence of conserved structural motifs, the telomerase specific motif T and reverse transcriptase motifs (magenta arrows), and variant-specific sequence characteristics (boxed). Polyploid variants are marked according to the parental origin of specific variants (Cs for *N. sylvestris*, O for *N. obtusifolia*, A for *N. attenuata*, W for *N. wigandiodes*, U for *N. undulata*, P for *N. paniculata* parent). Notably, *N. repanda*, *N. nesophila* and *N. stocktonii* (all Repandae) possess the *TERT*_O variant of *N. obtusifolia* with an in-frame mutation caused by a 102-nt-long deletion that occurs in the protein linker region that is suggested as important for telomerase catalytic function in humans. However, another 4-nt-long deletion within exon 9 results in an out-of-frame mutation in the same variant (stop codon is highlighted, translation of possible truncated variant is shown). (**B**) Comparison of three *TERT* variants from *N. tabacum* and *N. nudicaulis* shows the occurrence of *TERT*_Cs and *TERT*_D variants of *N. sylvestris* origin in both polyploids and mutations in the *N. nudicaulis TERT*_O variant. The *TERT*_O variant shows an amino acid transition D > N within motif C (two of three Asp residues essential for telomerase activity are depicted by triangles in motif C, and the remaining Asp residue is marked in motif A above) and a 4-nt-long deletion in the protein linker region proximal to motif D resulting in a stop codon. Structurally important regions from exon 9 (**A**) and exons 10, 11 and 12 (**B**) of *TERT* variants from polyploids and their progenitor diploids are shown, including nucleotide and protein consensus sequences (numbering of nucleotide sequence on top, full alignments are in Supplementary A2 and A3).

In contrast to the more ancient polyploids from the section Suaveolentes that originated from a single polyploidization event, followed by a reduction in genome size and chromosome number (2n ranges from 30 to 48), all other *Nicotiana* allopolyploids are 2n = 4x = 48, representing a doubling of the diploid chromosomal number for the genus (2n = 2x = 24) [31,32,35]. The monophyletic section Repandae originated from diploid progenitors closely related to *N. sylvestris* (the maternal genome donor) and *N. obtusifolia* (the paternal genome donor), and it comprises four species—*N. nudicaulis*, sister to and distinct (both morphologically and genetically) from the remaining three species—*N. repanda*, *N. nesophila* and *N. stocktonii* [32,33]. We captured two *TERT* sequences in cloned PCR products from *N. repanda*, *N. nesophila* and *N. stocktonii* and three *TERT* sequences in *N. nudicaulis* (Table 1, Supplementary A1, Figure 3). Using *TERT* variant-specific PCR primers for amplification of respective *TERT* variants and/or variant-specific restriction enzyme digestion of PCR products (Figure 2A–C), we confirmed the occurrence of all *TERT* variants identified in Repandae species, including the *TERT*_D variant detected in *N. nudicaulis* that could be assumed to be of *N. sylvestris* origin (Table 1). Moreover, using qPCR with *N. nudicaulis* genomic DNA as a template and specific primers (Supplemental Text S1, Table S3), we determined that the *TERT*_Cs, *TERT*_D and *TERT*_O variants occurred in the ratio 1:1:1. Interestingly, SNPs in the *N. obtusifolia*-like *TERT* sequence (*TERT*_O) differed between *N. nudicaulis* and three other species. The *TERT* _O variants from *N. repanda*, *N. nesophila* and *N. stocktonii* shared a striking 102 nt-long in-frame deletion within exon 9 (Figure 2A,B) that shortens the protein linker sequence between reverse transcriptase motifs 2 and A (details in Figure 3A and Supplementary A2). The *TERT*_O variants in these species also share a 4 nt long deletion within exon 9, resulting in a premature stop codon in proximity to motif A. In addition, we detected a stop codon within exon 11 of the *TERT*_O variant from *N. repanda* (MG242415, Supplementary A1), caused by a nucleotide transition from G to A. The *TERT*_O sequence from *N. nudicaulis* (MG242410) showed a nucleotide transition from G to A that would change the essential residue Asp (D) to Asn (N) within the motif C (details in Figure 3B and Supplementary A3) and the presence of several indels in exons 10, 11 and 12, resulting in out-of-frame mutations. These results suggest that currently existing *TERT* gene variants that originated from an ancient *N. obtusifolia* parent cannot produce a catalytically active TERT protein and could represent a pseudogene in all Repandae species.

#### 2.2.3. Nicotianae

**Figure 4:**
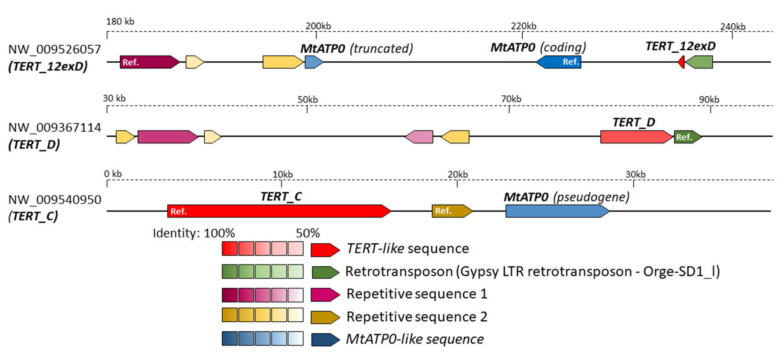
Arrangement of *TERT*-like sequences identified in the *N. sylvestris* genome assembly. Ancient *TERT* gene duplication is documented in contigs identified by BLAST search. The *TERT*_C variant represents a functional TERT copy. Two more contigs contained *TERT*-like sequences (*TERT*_D, *TERT*_12exD) that probably originated from *TERT* duplication and translocation because the *TERT*_12exD sequence stands for the 92 nt long region homologous to the end of exon 12 that is missing in the *TERT*_D variant. Mutual comparison of three *TERT* contigs in GEvo revealed the presence of the delta subunit of mitochondrial ATP synthase (*MtATP0*) that is often associated with *TERT*. Moreover, two repetitive sequences and retrotransposon Ogre-SD1_I that may be responsible for the split of *TERT*_D and *TERT*_12D sequences were identified. Sequence similarities (in %) are illustrated by a color scale relative to the reference sequence (Ref., 100%), as indicated.

The section Nicotianae is represented by *N. tabacum*, which originated from the most recent polyploidization event [31,40]. Three *TERT* variants (*TERT*_Cs, *TERT*_D of *N. sylvestris* origin and *TERT*_Ct of *N. tomentosiformis* origin) were characterized in detail experimentally [30,36]. To get a better insight into the origin and evolutionary fate of the *TERT*_D variant that is transcriptionally active and developmentally regulated in *N. sylvestris* and *N. tabacum* [36] but cannot produce a functional protein, we analyzed the *N. sylvestris* genome assembly (TW136) in silico. BLAST search identified three contigs (Figure 4). Two of them comprised sequences matching the previously identified (i) *TERT*_C variant (NW_009540950) and (ii) *TERT*_D variant (NW_009367114). A comparison of (i) and (ii) *TERT* variants revealed a *TERT*_D similarity with *TERT*_Cs, starting from the repetitive sequence region within intron 7 and ending within exon 12. (iii) In addition, we found a 92 nt region homologous to the 3′ end of exon 12 and also a putative 3′UTR region of *TERT* within contig NW_009526057 that apparently represents a part of the *TERT* sequence (Figure 4, *TERT*_12exD) that is missing in the *TERT*_D variant within the contig (ii). A BLAST search for the *TERT*_12D sequence in *N. tabacum* cv. TN90 genome assembly identified an unplaced genomic scaffold NW_015807891 with a sequence similar to *TERT*_12exD, thus indicating that *N. tabacum* retained all *TERT*-like sequences of *N. sylvestris* origin. To verify the number of *TERT*-like copies in silico, we analyzed raw data from SRA archives of *N. tabacum*, *N. tomentosiformis* and *N. sylvestris* genome sequencing projects. Quantification of a number of mapped SRA reads corresponding to respective *TERT* variants revealed equal occurrences of *TERT*_Cs:*TERT*_Ct:*TERT*_D in *N. tabacum*, and *TERT*_C:*TERT*_D variants in *N. sylvestris*, and a single *TERT* gene copy in the *N. tomentosiformis* (Table 2). In addition, we analyzed experimentally five available *N. sylvestris* accessions for the presence of *TERT*_C and *TERT*_D paralogs because previous genome sequencing [41] reported differences among accessions, including a burst of sequence amplification and/or homogenization. Using qPCR, we demonstrated the same relative abundance of *TERT*_C and *TERT*_D in all *N. sylvestris* accessions investigated, including the reference genome accession TW136 (Table 3, Supplemental Text S1).

### 2.3. Origin of the TERT Gene in Polyploid Sections Polydicliae, Rusticae and Undulatae

#### 2.3.1. Polydicliae

Two allopolyploid species, *N. quadrivalvis* and *N. clevelandii*, probably originated in polyploidization events that involved the same diploid parents [34,38,42]. The ancestor of *N. obtusifolia* functioned as a maternal genome donor and a progenitor of current *N. attenuata* as a paternal genome donor. We investigated the presence of *TERT* variants with different primer combinations covering exon 4 to exon 5, exon 9 and exon 10 regions (Tables S2 and S3). Experimentally, we detected a single *TERT* copy in both *N. quadrivalvis* and *N. clevelandii*, covering the genomic region from exon 4 to exon 5 that showed similarity to *TERT* from the *N. attenuata* lineage. Other primer combinations failed in *N. clevelandii*. However, during this study, transcriptomic SRA data from *N. clevelandii* became available, and a search for *TERT* variants uncovered the occurrence of two *TERT* variants that originated from both parental lineages (Table 1, deduced sequences used for analysis are in Supplementary A1).

#### 2.3.2. Undulatae and Rusticae

Two independent and relatively recent polyploidization events gave rise to *N. arentsii* (Undulatae) and *N. rustica* (Rusticae) (Figure 1). *N. arentsii* is an intrasectional polyploid that arose from related diploid parents closely related to *N. undulata* and *N. wigandioides* (all belonging to section Undulatae) [31,40]. Experimentally, we confirmed two distinct *TERT* variants in *N. arentsii* by sequencing of cloned PCR products and assigned them to their parental origins (Table 1, Supplementary A1). *Nicotiana rustica* was formed from diploid species closely related to *N. paniculata* (maternal genome donor) and *N. undulata* (paternal genome donor, [40]). Experimentally, we detected both parental *TERT* copies (*TERT*_P and *TERT*_U, respectively) in the *N. rustica* genome (Table 1, Supplementary A1).

### 2.4. Expression of TERT Variants in Nicotiana Polyploids

We further focused on the question of whether multiple variants of the *TERT* gene were transcribed (results summarized in Figure 1), especially in Repandae, where the *N. obtusifolia*-like *TERT* variant in all four polyploid species contains premature stop codons. We designed specific qPCR primers (Tables S2 and S3) spanning the 4-nt-long deletion to distinguish between parental *TERT* variants in *N. repanda*. Surprisingly, RT–qPCR analysis (Figure 2D) revealed that both variants, *N. sylvestris*-like (*TERT*_Cs) and *N. obtusifolia*-like (*TERT*_O), were transcribed at a comparable level in *N. repanda* seedlings. Using *TERT*-variant-specific primers in *N. nudicaulis*, RT–qPCR analysis revealed a higher transcript level of *TERT*_O in comparison with the *TERT*_Cs variant, while *TERT*_D transcripts were detectable but heavily under-represented. In *N. rustica* (Rusticae) seedlings, our RT–qPCR analyses showed comparable transcript levels of both *TERT* variants, *TERT*_P of maternal origin and *TERT*_U of paternal origin.

### 2.5. In Silico Analysis of N. sylvestris Genome Assembly Illustrates a Possible Evolutionary Scenario and the Origin of Subsequent Multiple TERT Loci

We detected both *N. sylvestris*-like *TERT*_Cs and *TERT*_D variants in *N. tabacum* [30] and *N. nudicaulis* experimentally (Table 1 and Figure 2C,D), but not in *N. benthamiana*. Looking at the time scale of polyploidization events (Figure 1) that gave rise to these species, this result suggests the occurrence of a *TERT*_D variant in ancient *N. sylvestris*, the progenitor of the section Repandae. The question is why the putative pseudogene variant is maintained in the current genome of *N. sylvestris*, as demonstrated in five accessions by qPCR (Table 3) and in its ancient polyploid offsprings.

**Figure 5:**
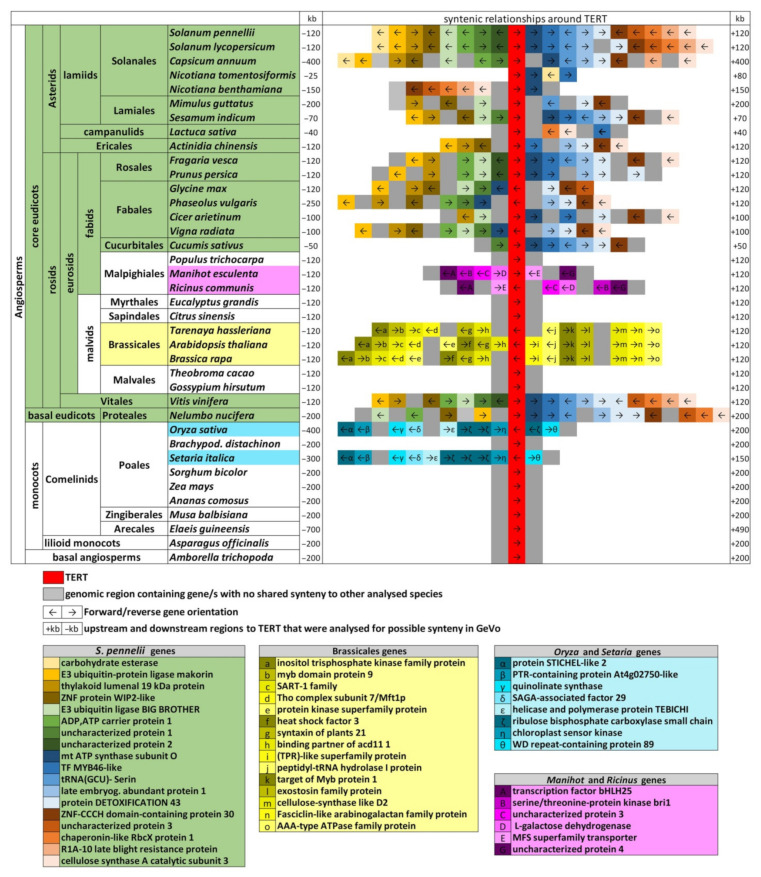
Graphical overview of *TERT* loci synteny across selected Angiosperms. Simplified presentation of *TERT* synteny is based on syntelogs visualized in GEvo among indicated plant *TERT* loci identified directly by SynFind in CoGe or by BLAST in NCBI genome databases. Dominant eudicot-like synteny (green) and three other types of shared gene synteny among close relatives, as indicated (yellow, cyan, purple), were identified across tested species. Source data used for GEvo analysis are listed in Table S5. SynFind parameters—algorithm:last; gene window size:30; minimum number of genes:5.

A comparison of three *TERT*-containing contigs from *N. sylvestris* (Figure 4) using the GEvo tool (https://genomevolution.org/coge/GEvo.pl) showed that the Ogre-SD1_I sequence was associated with both *TERT*_D and *TERT*_12exD sequences. RepeatMasker [43] classifies this highly repetitive sequence as an LTR/Gypsy retrotransposon. The position of Ogre-SD1_I suggests that *TERT*_12exD and *TERT*_D originated from the same ancient *TERT* locus. We presume that either retrotransposition- or transposon-facilitated ectopic recombination shattered the *TERT*_12exD and *TERT*_D sequences and transferred *TERT*_D to a completely new genomic locus. Another possibility is that the change within the ancestral *TERT* locus was mediated by a 1.3 kb long repeated sequence that is interspersed all over the *N. sylvestris* genome, including all three *TERT* contigs (Figure 4), and possibly serving as a hot spot for recombination [44]. Crucially, it is difficult to distinguish which of the copies, *TERT*_C or *TERT*_D, was derived from an ancestral copy because all three contigs are relatively short, and the *N. sylvestris* genome assembly is not complete. Moreover, *TERT*_12exD and *TERT*_C contigs show regions of sequence similarity downstream of the *TERT*s, corresponding to the gene for mitochondrial ATP synthase subunit delta (*MtATPO*) (Figure 4). A copy of *MtATPO* within the *TERT*_C contig is annotated as a pseudogene, while the *TERT*_12exD contig contains two copies of the *MtATPO* gene, one representing a putative functional copy while the other (inverted) copy is a pseudogene. *MtATPO* is usually a single-copy gene, and it is often associated with *TERT* within land plants, including *N. tomentosiformis* (contig NW008896550.1, compare in Figure 5). Thus, the presence of a functional *MtATPO* gene copy may be the reason the genomic loci containing the *TERT*_12exD variant were retained (see Supplemental Text S1).

### 2.6. Genomic TERT Loci Analysis Defines Ancestral Synteny within Flowering Plants

The turbulent history of *TERT* copies within *Nicotiana* polyploid genomes directed our attention to the question of the genomic arrangement of the *TERT* locus. *TERT*-containing contigs in *N. sylvestris* are short and/or gene-free (Figure 4), so there are no checkpoints for comparison of microsynteny, and only short *TERT*-containing contigs could be analyzed in *N. tomentosiformis* (Figure 5), the progenitor diploid of *N. tabacum*. Thus, we focused on a comprehensive analysis of the gene order (syntelogs) of *TERT* neighboring genes across representative plant genomes (Table S5). Syntelog is a special case of gene homology where sets of genes are derived from the same ancestral genomic region. This homology may arise from speciation events or through whole or partial genome duplication events. Initially, we asked whether there was any syntelog of the genes neighboring *TERT* within the Solanaceae species. Then we examined species from plant clades closely or distantly related to the Solanaceae family. Using SynFind [45] and the *Solanum penellii* genome assembly as a reference, we identified syntelogs among Solanaceae species tested, and these were subsequently visualized by GEvo. *TERT* microsynteny similar to *S. penellii* was found in 18 of 49 genomes analyzed, including ancient polyploids and evolutionarily distant angiosperms (Figure 5). In addition, many *TERT* loci showed co-linearity. These were representatives of large taxonomic groups (Asterids and Fabids) and species representing basal clades—*Vitis vinifera* (Vitales, basal for rosids), and *Nelumbo nucifera* (Proteales, basal eudicots), but not *Amborella* (early diverging angiosperm lineage) or green algae, suggesting that the detected *TERT* microsynteny may have originated in eudicots subsequent to the divergence of the *Amborella* lineage. Interestingly, 16 of 18 genes neighboring *TERT* in *S. penellii* were almost co-linear within the *Vitis TERT* locus, and 14 of 18 genes were shared in the *Nelumbo TERT* locus. Thus, with the exception of the carbohydrate esterase gene that is exclusive to the *TERT* loci in Solanales, these genes occurred within the *TERT* locus in species grouped by ancestral eudicot-like microsynteny (green, Figure 5). This microsynteny of the *TERT* locus was secondarily lost in several plant lineages that show the *TERT* gene translocated into completely different genomic regions. We reciprocally compared these novel *TERT* genomic regions to determine whether any other *TERT* syntelogs were evolutionarily conserved within Angiosperms. We identified *TERT* microsynteny among closely related species from Brassicales and Malpighiales within eudicots (Figure 5). With the exception of two species from Poales, no other conserved *TERT* syntelogs were detected among monocots or within other particular eudicot species/clades. Thus, novel genomic *TERT* loci show microsynteny restricted to closely related species.

**Figure 6:**
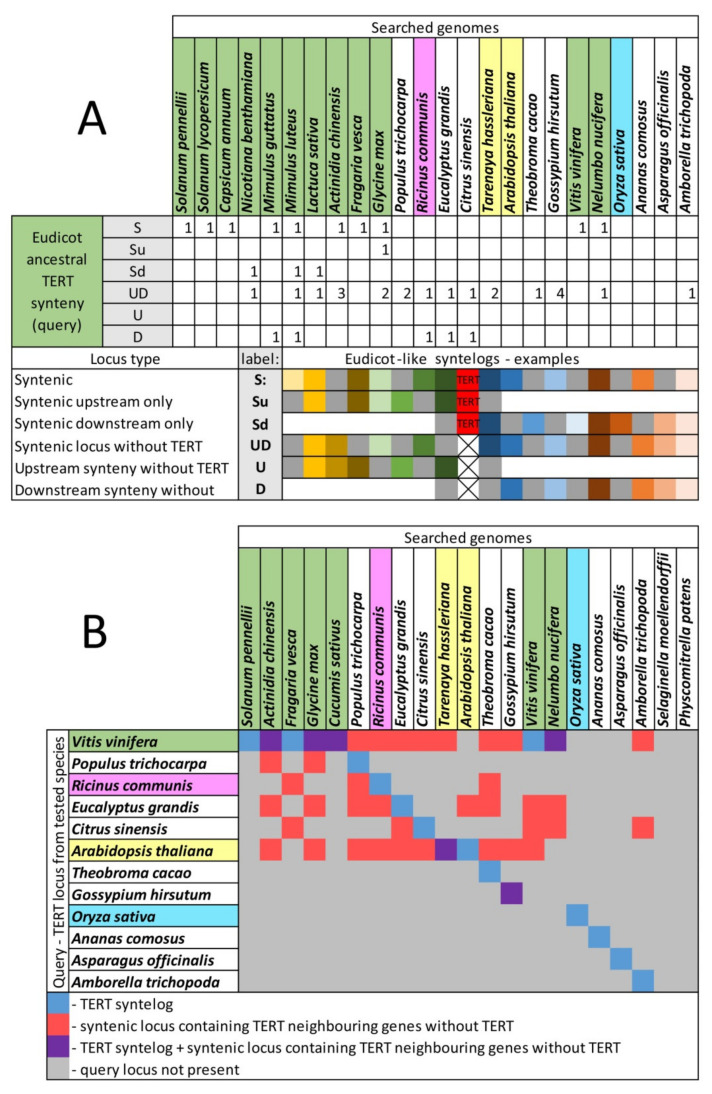
Occurrence of *TERT* syntelogs in Angiosperms. (**A**) Detailed analysis of the eudicot-like type of synteny (represented by *Vitis* syntelog as a query) in indicated genomes shows the presence of syntenic regions with/without the *TERT* gene in the majority of investigated eudicots and *Amborella*. The number of syntelogs and synteny categories are shown for each species. (**B**) The occurrence of conserved syntenic regions corresponding to the species-specific *TERT* query was investigated in representative genomes. Co-occurrence of syntelogs in more species suggests an ancient origin of target loci that accommodated *TERT* in current species. Analyses were carried out using CoGe, GEvo and SynFind. SynFind parameters—algorithm: last; Gene window size: 30; minimum number of genes: 5.

We then asked how conserved was the ancestral eudicot locus that accommodated the *TERT* gene with respect to its occurrence in current genomes. To answer this question, we searched for the presence of genomic loci involving only syntenic genes neighboring *TERT*, i.e., with eudicot-like microsynteny (green, Figure 6A). We identified such original loci (without a *TERT* gene) in eudicots (except *A. thaliana*) and *Amborella*, but not in monocots or representatives of other basal clades (*Physcomitrella*, *Selaginella*, green algae). Moreover, several genomes showed the occurrence of more loci with eudicot-like microsynteny, either complete (UD, Figure 6A) or comprising downstream neighboring gene pairs (D, Figure 6A).

A reciprocal search was then carried out using syntenic genes neighboring *TERT* in novel genomic loci that accommodated *TERT* in current genomes (termed here as, e.g., *Arabidopsis*-like, *Populus*-like, *Oryza*-like synteny, Figure 6B). This identified these loci in eudicots, but not in monocots and basal clades (*Physcomitrella*, green algae). For example, the syntelog derived from the *TERT* locus of *Citrus sinensis* (*Citrus*-like) occurred in *Amborella*, *Nelumbo*, *Vitis*, *Eucalyptus* and *Fragaria* genomes. Within the *Citrus sinensis* genome, loci with the eudicot-like (represented by *Vitis*-like query) and *Arabidopsis*-like synteny were present. Interestingly, *Theobroma cacao* (Malvales) that harbors the species-specific *TERT* locus contained loci with eudicot-like, *Ricinus*-like, *Eucalyptus*-like and *Arabidopsis*-like synteny, but loci with *Theobroma*-like synteny did not occur in the other genomes investigated.

### 2.7. The Occurrence of TERT Homologs in Model Species Illustrates a Possible Origin of TERT Variants

**Figure 7:**
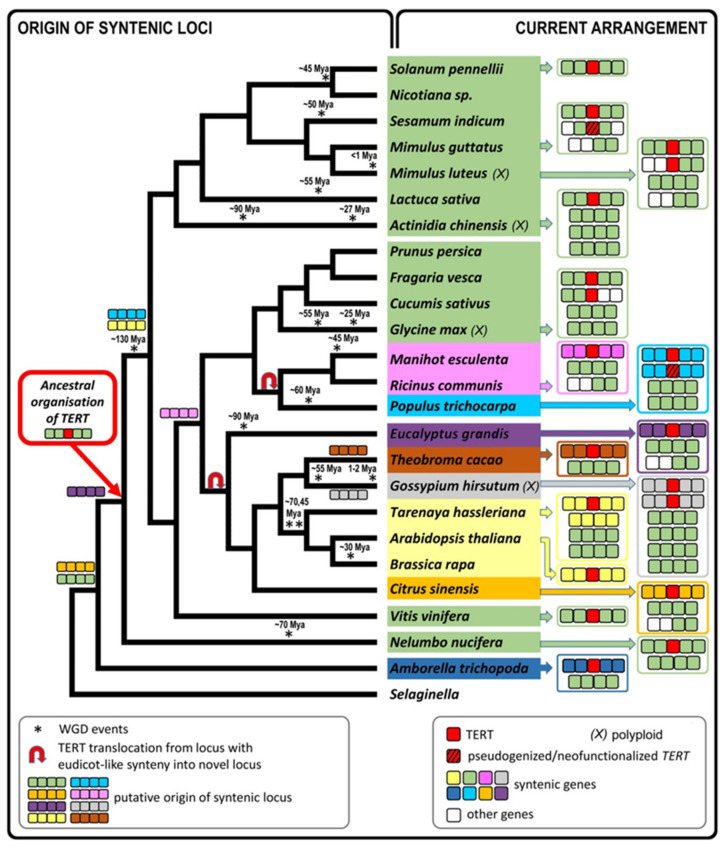
Origin of syntenic *TERT* loci in angiosperms. An ancestral locus with eudicot-like synteny that is present in the *Amborella* genome adopted the TERT gene in basal eudicots (*Nelumbo*). This ancestral locus with eudicot-like synteny occurs within the genomes of eudicots with the exception of the model plant *Arabidopsis thaliana* (for simplicity, diagrams on right panels show occurrence and arrangement of loci with eudicot-type synteny and with a specific synteny if present). The TERT gene was translocated several times into novel loci with a conserved synteny observed in current species (termed here as *Citrus*-like, *Populus*-like, *Eucalyptus*-like, *Ricinus*-like, *Arabidopsis*-like syntelogs) that had already occurred in ancestors (nodes depicting synapomorphic relationships of specific synteny-types and thus the putative origin of ancestral syntenic loci are shown above respective phylogeny nodes). As an exception, Malpighiales (*Theobroma*, *Gossypium*) show the TERT gene translocated into novel species-specific loci. These genomes nevertheless still contain the ancient loci with conserved synteny (details in Figure 6). *TERT* is mostly maintained as a single copy gene, but polyploid species can tolerate more copies (*M. luteus*, *G. max*, *G. hirsutum* are shown as representatives). Copies of genomic loci with the original synteny remain present after *TERT* gene elimination, e.g., in *Actinidia chinensis*, where it is difficult to distinguish which of the ohnologous loci (ohnologs = paralogs derived by WGD) have lost their TERT gene copy (see Supplemental Text S1, Figure S2). Phylogeny was adapted from APG IV [52], WGDs were mapped according to [7] in eudicots, and according to [53] in *Actinidia*.

The same questions about genomic arrangement of the *TERT* locus and number of gene copies were asked about polyploid model species *Glycine max*, *Gossypium hirsutum*, *Brassica napus*, *Camelina sativa*, *Mimulus luteus* and *Actinidia chinensis* (Figure 6, Supplemental Text S1, Table S5, summarized in Figure 7 and Figure S2). 

*Actinidia* (x = 29, Ericales) is a paleotetraploid derived from an ancestor with x = 14 [46,47] that resembles the ancient *N. benthamiana* speciation, with changes in chromosome number in the section Suaveolentes. One *TERT* locus and three additional loci with eudicot-like synteny were present in the *A. chinensis* genome (Figure 6A). *Glycine max* (Fabales) is a paleopolyploid with a highly duplicated genome, where nearly 75% of the genes are present in multiple copies [48]. The *TERT* gene is no exception, and we identified two loci on chromosomes 15 and 8 (LOC100790649 and LOC100776816, respectively) that contain functional copies of TERT. The *TERT* locus on chromosome 15 shares a full eudicot-like synteny, while the arrangement of the *TERT* locus on chromosome 8 was similar to the eudicot-like synteny only in the region upstream of syntenic *TERT* (S and Su, respectively, Figure 6A). Additionally, two regions with similarities to eudicot-like synteny, but without a copy of the *TERT* gene, were located on chromosomes 12 and 13 (UD, Figure 6A). The occurrence of eudicot-like synteny in *Mimulus luteus* (Lamiales) resembles *Glycine max* with two *TERT* loci (S and Sd) and two additional loci without a *TERT* gene (UD and D, Figure 6A). The number of *TERT* gene copies in Brassicaceae reflects ploidy level with 2 and 3 *TERT* genes in tetraploid *Brassica napus* and hexaploid *Camelina sativa*, respectively (Table S5). However, one of the *TERT* copies in *C. sativa* is a putative pseudogene. The tetraploid genome of *Gossypium hirsutum* that originated ca. 1–2 Myr ago [2,49] contains two *TERT* genes and four additional loci without a *TERT* gene that share eudicot-like microsynteny (Figure 6A).

Intriguingly, during in silico identification of *TERT* loci, we noticed that the analyzed diploid species, *Populus trichocarpa, Mimulus guttatus*, *Amborella trichopoda*, and *Vigna radiata*, contained more than one *TERT*-like sequence, which could illustrate maintenance of *TERT* variants (details in Supplemental Text S1, Table S5, Figure S3). In *Amborella trichopoda* (Figure S3A), the search for *TERT* revealed the full-length *TERT* gene (LOC18433477) and a truncated *TERT*-like sequence (LOC18443854) covering 208 amino acids from the N-terminal part of the TERT protein. However, the origin of the truncated *TERT*-like sequence is unclear (see Supplemental Text S1). In *P. trichocarpa, M. guttatus* and *V. radiata*, the additional *TERT-like* copy could be classified as a pseudogene or may function as an ncRNA (Figure S3B–D).

## 3. Discussion

To test experimentally and in silico *TERT* gene balance following ancient polyploidization events, we identified and characterized *TERT* copies in genomes of polyploid *Nicotiana* species and representatives of their diploid progenitors. We also investigated the expression of *TERT* variants identified in the polyploids using RT–qPCR. We found that the *N. sylvestris* progenitor was a very successful parent of sections Suaveolentes, Repandae and Nicotianae because the *TERT*_Cs variant of *N. sylvestris* origin was identified in all polyploid genomes investigated (Figure 1), and high levels of its transcripts were detected. Moreover, an additive occurrence of *TERT* copies observed in *N. tabacum* and *N. nudicaulis* suggests that gene/genome duplication resulting in the formation of *TERT*_C and *TERT*_D variants in *N. sylvestris* had occurred at least before the formation of the section Repandae. The *TERT*_D transcripts were detectable but heavily under-represented in *N. nudicaulis* (Figure 2D), similar to *TERT*_D expression in *N. sylvestris* and *N. tabacum* [30,36]. In contrast to the success of the *N. sylvestris* progenitor, the *TERT*_O variant of *N. obtusifolia* origin was pseudogenized in all four polyploid species from Repandae. A 102-nt-long in-frame deletion within exon 9 would shorten the linker region between motif 2 and motif A, including protein motif GSSVF that is well-conserved in plant TERTs. This region, termed as motif 3 in human TERT, was found to be crucial for telomerase catalytic functions [50]; however, its absence is not the only problem in *TERT*_O variants. Various indels found across *TERT*_O variants from Repandae would result in out-of-frame mutations, and interestingly, a nucleotide transition found within motif C of *N. nudicaulis TERT*_O would disrupt one of three Asp residues that are essential for the catalytic function of any telomerase [28,29]. However, mRNA levels of the *TERT*_O variant revealed expression comparable to the *TERT*_Cs variant in *N. nudicaulis* and *N. repanda* (Figure 2D). Comparable transcript levels of parental *TERT* variants coding for the functional TERT protein were detected in the relatively young polyploid, *N. rustica* (*TERT*_P and *TERT*_U, Figure 2D), and similarly in *N. tabacum* [36].

Our experimental analyses were accompanied by an in silico approach to answering the question on the origin and fate of the *TERT*_D variant in the *N. sylvestris* genome and, for a wider perspective, in other polyploid plant genomes. Our experimentally estimated ratio 1:1 of *TERT*_C and *TERT*_D gene copies in five *N. sylvestris* accessions was confirmed by in silico analysis of raw data from the *N. sylvestris* genome sequencing project (Table 2). Moreover, we identified a part of the *TERT*_D variant sequence (*TERT*_12D) associated with high-copy repetitive sequences, and the *MtATPO* gene, within a novel genomic locus in the *N. sylvestris* genome (Figure 4). An unplaced genomic scaffold arranged similarly to the *TERT*_12D locus was identified in *N. tabacum*, suggesting that an ancestral split of the *TERT*_D copy had occurred at least before the formation of *N. tabacum*. There is no information about a species-specific WGD event or an additional genome donor in *N. sylvestris*, but the increase in transposable elements and repeats was reported [41]. Moreover, activation of transposable elements was observed as a stress response to genome instability that may have been caused by a polyploidization event or environmental stress [41,51]. We presume, therefore, that the ancestral *TERT*_D locus (including *TERT*_12exD and *MtATPO*) originated as a result of gene/segment duplication of the *TERT*_C (plus *MtATPO*) locus or vice versa (Supplemental Text S1, Figure S2). Both loci were pseudogenized—the *TERT*_C locus within the *MtATPO* region and the *TERT*_D locus within the *TERT* region—and later, the *TERT*_D locus was split and translocated by Ogre/SD1-I. Currently, the mutual positioning of *TERT*_C, *TERT*_D and *TERT*_12exD within the genome of *N. sylvestris* is not known; however, similar scenarios could have resulted in pseudogenization and/or neofunctionalization of an additional *TERT* gene copy that we found in diploid species *Populus trichocarpa*, *Vigna radiata* and *Mimulus guttatus*. These *TERT-like* sequences may illustrate possible scenarios leading to the formation of *TERT* pseudogene variants in *Nicotiana* and the progression of gene elimination after gene/genome duplication: (i) A large-scale segment/genome duplication event had created an additional *TERT* locus, presumably encoding a *TERT* pseudogene on chromosome 1 in *Populus*, (ii) two *TERT* copies placed on the same scaffold in *Mimulus*. (iii) A completely different arrangement comprising an additional *TERT* variant of *Vigna radiata* that is formed by two adjacent inverted copies of exon 9 of *TERT*, and this *TERT*-like sequence was annotated as ncRNA (summarized in Figure S2). Multiple *TERT* copies were present in some, but not all polyploid species investigated, and toleration of more *TERT* copies after young polyploidization events is obvious (Figure 7).

Regarding the origin and evolution of the *TERT* loci in eudicots, comparison of eudicot phylogeny relationships [52] with the occurrence of syntenic loci that adopted the *TERT* gene demonstrated ancient synapomorphies, i.e., loci preserved in current genomes are assumed to have been present in their most recent common ancestor (nodes are depicted in Figure 7). The eudicot-like synteny locus emerged in early eudicots (*Amborella*) and adopted the *TERT* gene later in the ancestral parent of *Nelumbo*. The original *Amborella TERT* locus was probably fragmented. Another translocation of the *TERT* gene into novel loci grouped in all investigated malvids and Malphigiales (in fabids), and further translocations to other loci, took place later on. Interestingly, in several cases, we detected a translocation into loci that had already existed in ancestral genomes for a long time, e.g., the locus with the *Citrus*-like synteny originated in early eudicots, as assumed from the locus synapomorphy. The first *TERT* translocation from a locus with eudicot-type synteny was not caused by locus fragmentation because these loci occur in current eudicots (Figure 6B), the only exception being *A. thaliana*. Moreover, destabilization of the *TERT* position within the eudicot-like synteny locus was probably not caused by gene rearrangement because the predicted *TERT* gene structure with 10 exons is specific for *Populus* and does not occur in other Malphigiales, and *TERT*s with 13 exons were found in Solanaceae [30] that share eudicot-like synteny. Thus, it could be speculated that the successful *TERT* translocation event was more likely into target loci that show ancient synapomorphy (Figure 7). The only exceptions from this observed pattern are the *Theobroma* and *Gossypium* loci that were not syntenic to other genomes. This could indicate that these species-specific translocations are relatively recent.

In conclusion, our results show that natural *Nicotiana* polyploids tolerate more *TERT* copies and, similarly to other polyploid genomes investigated, retention of various copies is obvious in species formed by young polyploidization events. A comparison of *TERT* locus arrangement in current genomes suggests that the *TERT* gene was placed in a conserved locus that became advantageous following the emergence of basal eudicots (Figure 7). The gene was relocated later in several plant groups where only a narrow syntenic relationship restricted to closely related species could be found. Various evolutionary scenarios took place in ancestral genomes with multiple *TERT* copies resulting in elimination, pseudogenization and/or fragmentation, and neofunctionalization of novel *TERT* copies that could also illustrate the origin and fate of *N. sylvestris* and polyploid *Nicotiana TERT* variants (Figure S2).

## 4. Materials and Methods

### 4.1. Isolation of Plant Material, Genomic DNA and RNA

*Nicotiana* species and their accessions are listed in Table S1. *N. sylvestris* (accessions 934750005, TW136), *N. obtusifolia*, *N. nesophila* and *N. stocktonii* were kind gifts from Prof. Marie-Angèle Grandbastien (INRA, France). *N. sylvestris* (accession A04750326), *N. repanda*, *N. nudicaulis*, *N. paniculata*, *N. undulata*, *N. rustica*, *N. clevelandii*, *N. attenuata* and *N. alata* were gifts from Prof. Andrew Leitch (Queen Mary University London, UK). *N. sylvestris* (accessions 626, Ducretet 101–268), *N. quadrivalvis*, *N. wigandoides* and *N. arentsii* were purchased from Imperial Tobacco Bergerac (France). Plants were grown in growth chambers under conditions of 16 h light, 22 °C and 8 h dark, 19 °C, illumination 150 µmol·m^−2^·s^−1^. DNA for qPCR experiments was isolated from plant leaves, according to Dellaporta et al. [54]. Total RNA was isolated from seedlings or young leaves using NucleoSpin^®^ RNA kit (Macherey-Nagel, Dueren, Germany). RNA was purified by DNaseI treatment (Turbo DNA-free, Life Technologies), and its integrity was checked by electrophoresis on a 1% (*w/v*) agarose gel. RNA concentration was measured using a spectrophotometer (Nanodrop).

### 4.2. PCR Amplification of TERT Sequence Variants

For detection of variant *TERT* sequences in a single PCR reaction (25 µL), we used 200 ng of genomic DNA from various *Nicotiana* species as a template, KAPA Taq DNA Polymerase (Kapa Biosystems, Wilmington, MA, USA) and primer combinations listed in Tables S2 and S3. Thermocycling conditions for PCR reactions were as follows: 1 min at 95 °C, 35 cycles of 15 s at 95 °C, 15 s at 56 °C, 2 min at 72 °C, final extension 7 min at 72 °C. PCR products were checked on a 1% (*w/v*) agarose gel. For sequencing (Macrogen **Europe B.V., Amsterdam, Netherlands**), we purified PCR products using a QUIaquick PCR purification kit (Qiagen, Valencia, CA, USA). In the case of *N. repanda*, *N. nesophila* and *N. stocktonii*, PCR products were purified from an agarose gel using a QUIaquick gel purification kit). Alternatively, PCR products were cloned into the pCRIITOPO vector using a TOPO TA cloning kit (Invitrogen, Carlsbad, CA, USA) and sequenced to analyze individual *TERT* variants. Representative sequences were submitted to GenBank (all accessions are specified in Table S3 and Supplementary A1). All primers are listed in Table S2.

### 4.3. Quantitative PCR and RT–qPCR

The number of *TERT* copies in genomes of *N. sylvestris* accessions was investigated using qPCR and *TERT* variant-specific primers under qPCR conditions described in [36].

For RT–qPCR, we prepared cDNA from 2 μg of total RNA according to the manufacturer’s protocol using M-MuLV reverse transcriptase (New England Biolabs, Ipswich, MA, USA) and random nonamers (Sigma, Saint Louis, MO, USA). Quantification of the relative transcription levels of the *TERT* paralogs in 10 day-old seedlings of *N. rustica, N. repanda* and *N. nudicaulis* was performed in three technical replicates using *TERT* variant-specific primers and KAPA SYBR FAST qPCR master mix (Kapa Biosystems) in a Rotorgene 6000 cycler (Qiagen, Valencia, CA, USA). For comparative quantification of paralogous *TERT* transcripts in *N. repanda* and *N. rustica*, we optimized qPCR conditions to reach the same efficiency for *TERT* variant-specific reactions (Table S3). Optimized qPCR conditions were as follows: 5 min at 95 °C, 40 cycles of 5 s at 95 °C, 20 s at 62 °C, 15 s at 72 °C, final extension 3 min at 72 °C. RT–qPCR for three *TERT* variants in *N. nudicaulis* was performed using specific primer combinations and EliZyme Green MIX AddROX (Elisabeth Pharmacon, Brno, Czech Republic) in a Rotorgene 6000 cycler under the following conditions: 3 min at 95 °C, 45 cycles of 5 s at 95 °C, 20 s at 61 °C, 15 s at 72 °C, final extension 3 min at 72 °C. The proportion of *TERT* transcripts in allopolyploids were calculated by the delta Ct method [37] and normalized according to PCR efficiency determined from calibration curves.

### 4.4. In Silico Identification of TERT Variants in Nicotiana Species

*TERT* gene sequences were constructed in silico using genome assembly data from *N. attenuata* and *N. obtusifolia* (accessions: GCA_001879085.1 and GCA_002018475.1, respectively) and transcriptomic SRA data from *N. noctiflora* (SRR2106514) and *N. clevelandii* (SRX3866257).

For identification of *TERT* variants in *N. benthamiana*, we carried out a BLAST (BLASTn) search in genome assembly (Genome ID: 20448, [39] using full-length CDS of *N. sylvestris TERT* (LOC104217220) as a query. Subsequently, we analyzed raw whole-genome NGS data (kindly provided by Prof. Aureliano Bombarelly and Prof. Gregory Martin, leaders of the BTI *Nicotiana benthamiana* genome Project): two PE libraries comprising 4 files, 2 × 100 nt; 500 bp insert size; 16 Gb compressed file size/each corresponding to ≥30× genome coverage. We built a nucleotide BLAST database on MetaCentrum using BLAST+ command-line applications. *TERT-like* reads were identified by BLAST and mapped back to the query using Geneious software (Biomatters Ltd., Auckland, New Zealand). As a proof of concept, this approach was tested on publicly available genomic SRA data from *N. tabacum*, *N. tomentosiformis* and *N. sylvestris* (SRX338107, ERX248865 and ERX248848, respectively). Identification of similarities among *TERT* contigs from *N. sylvestris* and repetitive elements was performed using GEvo with/without masking of non-CDS regions and validated manually. To determine the parental origin of *TERT* variants in polyploids, cloned fragments and/or corresponding sequences reconstructed from genomic/transcriptomic databases were aligned with *TERT* sequences from their progenitor diploids. Alignments were generated, and pairwise % identities (shown in Table 1) were calculated using Geneious software.

### 4.5. Analysis of Gene Synteny of the TERT Locus within Angiosperms

We selected 49 representative species (Table S5) across the Angiosperm phylogeny and basal clades with deeply sequenced and well-annotated genomes from CoGe [55] or publicly available at GeneBank. *TERT*-containing genomic regions were identified using FeatView (in CoGe genomes) or BLAST (www.ncbi.nlm.nih.gov) for subsequent syntenic analysis. The syntenic analysis of the *TERT* loci (Figure 5) was performed in two steps. First, we used the SynFind tool [45] as a screening approach for the existence of syntenic relationships between the tested *TERT* genomic region from one species against genomes (CoGe) of other Angiosperms. SynFind identified syntenic regions using a *TERT* locus query from one genome against any set of genomes. Then *TERT* genomic regions from species that shared gene synteny were grouped and analyzed using the GEvo tool in CoGe [55]. GEvo served for comparison of multiple large genomic regions, identification and visualization of local BLAST hits. The GEvo setup for analysis of synteny, i.e., sequence masking, was: non-CDS—used for reference sequence; algorithm: BLASTz; word size: 8; gap start penalty: 400; score threshold: 3000; minimum HSP length: 40. Information necessary for SynFind and GEvo analyses including genome accessions and *TERT* loci) are listed in Table S5. Identification of species-specific loci containing *TERT* neighboring genes, but not the *TERT* gene in other species, was performed using SynFind (setup: comparison algorithm—last; gene window size—30; minimum number of genes—5). We used representative *TERT* loci for each synteny type (Figure 5, Supplemental Text S1, Table S5) as a query against the genomes tested. SynFind outputs were further analyzed in GEvo for visualization and manual syntelog classification.

## Figures and Tables

**Table 1:** Origin of telomerase reverse transcriptase (*TERT*) variants in polyploid *Nicotiana* species determined by sequence similarity with representative progenitor diploids.

Allopolyploids	GeneBank Accessions	Sequence Similarity [%]				Analyzed Region ^1^
		Maternal Parent		Paternal Parent		
**SUAVEOLENTES**		***N. alata***	***N. noctiflora*^2^**	***N. syl. C var.***	***N. syl. D var.***	
*N. benthamiana*	NbS000104	96.3	n.a.	**97.5**	n.a.	exon 4 to 5
	27g0116.1	n.a.	96.1	**97.6**	93.4	exons 10, 11, 12
**REPANDAE**		***N. syl. C var.***	***N. syl. D var.***	***N. obtusifolia***		
*N. repanda*	MG242402 ^1^	95.9	91.6	**97.4**		exon 9
	MG242403 ^1^	**97.9**	92.4	96.4		exon 9
*N. stocktonii*	MG242407 ^1^	95.6	91.7	**97.6**		exon 9
	MG242408 ^1^	**98.6**	93.1	97.0		exon 9
*N. nesophila*	MG242405 ^1^	95.2	91.6	**97.0**		exon 9
	MG242406 ^1^	**98.5**	92.9	96.9		exon 9
*N. nudicaulis*	MG242409 ^1^	**98.6**	94.3	94.4		exon 10 to 12
	MG545647 ^1^	92.8	**94.8**	91.6		exon 10 to 12
	MG242410 ^1^	94.2	93.3	**96.3**		exon 10 to 12
**POLYDICLIAE**		***N. obtusifolia***		***N. attenuata***		
	MG242422 ^1^	94.3		**99.3**		exon 4 to 5
*N. clevelandii*	var1 ^2^	97.3		**98.9**		exon 9 ^2^
	var2 ^2^	**99.2**		97.3		exon 9 ^2^
*N. quadrivalvis*	MG242423 ^1^	94.9		**98.6**		exon 4 to 5
**ARENTSII**		***N. undulata***		***N. wigandiodes***		
*N. arentsii*	MG242418 ^1^	**99.5**		98.4		exon 9
	MG242419 ^1^	98.8		**99.8**		exon 9
RUSTICA		*N. paniculata*		*N. undulata*		
*N. rustica*	MG242413 ^1^	**100.0**		98.2		exon 9
	MG242414 ^1^	98.2		**99.8**		exon 9

^1^ all sequences cloned in this work are in Supplementary A1, including corresponding sequences cloned from progenitor diploids; ^2^ regions mapped to raw RNAseq data or extracted from genome assembly (Supplementary A1).

**Table 2:** Number of *TERT* gene copies in *Nicotiana* species determined in silico.

Species/Genome Dataset Accession	Total No. of *TERT* Reads	Expected Genome Coverage (Depth)	No. of Detected *TERT* Variants	Read Counts Corresponding to Known *TERT* Variants			Ratio of *TERT* Variants in Genome
*N. tabacum* SRX338107	1259	35×	3	*NtTERT_Cs*	*NtTERT_D*	*NtTERT_Ct*	1:1:1
				425	424	410	
*N. sylvestris* ERX248848	644	26×	2	*NsTERT_C*	*NsTERT_D*		1:1
				332	312		
*N. tomentosiformis* ERX248865	203	15×	1	*NtomTERT*			-
				203			
*N. benthamiana* (raw data from [39])	286	20×	1	*NbenTERT*			-
				286			

**Table 3:** Number of *TERT* copies in *N. sylvestris* accessions determined by qPCR.

*N. sylvestris* Accession	Ct (±SD)			∆Ct (C-C1/2×)	∆Ct (C-D)	C:D Ratio
	Ns*TERT*_C	Ns*TERT*_C 1/2× (Control)	Ns*TERT*_D			
A04750326	16.41 (±0.036)	16.91 (±0.085)	16.41 (±0.065)	−0.5	0.00	1:1
934750005	16.65 (±0.052)	17.12 (±0.043)	16.59 (±0.049)	−0.47	0.06	1:1
ITB626	17.65 (±0.067)	18.12 (±0.051)	17.66 (±0.035)	−0.47	−0.01	1:1
TW136	17.25 (±0.02)	17.68 (±0.015)	17.13 (±0.043)	−0.43	0.12	1:1
Ducrettet 101-268	17.31 (±0.023)	17.95 (±0.063)	17.26 (±0.08)	−0.64	0.05	1:1

## Data Availability

Sequences were submitted to Genbank (NCBI, www.ncbi.nlm.nih.gov) under accession numbers MG242402- MG242425 and MG545647.

## Supplementary Materials

Supplementary materials can be found at https://www.mdpi.com/1422-0067/22/4/1783/s1.

## Abbreviations

BLASTn	Basic local alignment search tool, nucleotide to nucleotide
LTR	Long terminal repeat
MtATPO	Mitochondrial ATP synthase subunit delta
NGS	Next-generation sequencing
qPCR	Quantitative PCR
SNP	Single nucleotide polymorphism
SRA	Short read archive
TERT	Telomerase reverse transcriptase
WGD	Whole-genome duplication
