# Supplementary material for: Origin and Fates of TERT Gene Copies in Polyploid Plants
Source: Int J Mol Sci. 2021 Feb 11;22(4):1783. doi: 10.3390/ijms22041783 (PMC7916837; doi:10.3390/ijms22041783)

Internal Journal of Molecular Sciences

Supplementary material

Origin and fates of the *TERT* gene copies in polyploid plants

Petr Fajkus[1], Vratislav Peška[1], Jiří Fajkus[1,2,3*] and Eva Sýkorová[1*]

[1] Institute of Biophysics of the Czech Academy of Sciences, Královopolská 135, CZ-61265 Brno, Czech Republic

[2] Laboratory of Functional Genomics and Proteomics, NCBR, Faculty of Science, Masaryk University, Kamenice 5, CZ-62500 Brno, Czech Republic

[3] Mendel Centre for Plant Genomics and Proteomics, CEITEC, Masaryk University, Kamenice 5, CZ-62500 Brno, Czech Republic

* To whom correspondence should be addressed. Tel: +420 541 517 199, e-mail: **evin@ibp.cz**

Supplementary Text S1. Experimental and *in silico* analyses

Analysis of TERT variants identified in *Nicotiana* species.

As an illustration of sequence relationship between *TERT* variants in polyploid species and the *TERT* gene in respective parental copies, we adopted an approach alternative to traditional calculation of sequence similarity (%, **Table 1**). Here we show supplemental analysis of SNPs (similarity as X/Y, **Table S4** and illustrated in **Figure S1**). As first, we compared *TERT* sequences of amplified (or identified *in silico*) from respective progenitor diploids and we identified a set of SNPs (single nucleotide polymorphisms) that indicate differences between these parental copies (Y, **Table S4**). Then we compared the sequences of *TERT* variants from polyploid and calculated the number of individual SNPs homologous to each of the parental SNPs (X, **Table S4**, analysis illustrated in **Figure S1)**. For example, we examined the similarity of the *N. benthamiana TERT* sequence to a subset of parental-specific SNPs, (i) between sequences of *N. sylvestris* (variant C) and *N. alata* we found 19 SNPs within a 591 bp long genomic region from exon 4 to exon 5, and (ii) *N. sylvestris* (variant C) and *N. noctiflora* we found 30 SNPs within a 1112 bp long region combining exons 10, 11, and 12. Detailed comparison of these individual SNPs showed the *TERT* sequence of *N. benthamiana* shared most SNPs with *N. sylvestris TERT* in both analysed regions (i) and (ii) (14 of 19 parental SNPs from exon 4 to exon 5 region, and 20 of 30 parental SNPs from combined exons 10, 11, and 12, respectively, **Table S4**). This result corresponds to sequence similarity (in %) shown in **Table 1**. Another example is a comparison of *N. sylvestris* (variant C) and *N. obtusifolia* sequences from exon 10 to exon 12 that contained 56 SNPs shown in the analysis of *N. nudicaulis* variants. Comparisons with *TERT* variants that were amplified from *N. nudicaulis* showed that (i) in one cloned variant (represented by Genbank accession MG242409), 42 from 56 parental SNPs were identical with the *N. sylvestris* sequence and only 2 SNPs were shared with *N. obtusifolia,* thus the sequence MG242409 is clearly of *N. sylvestris* origin; (ii) in another cloned variant (MG242410), 7 from 56 parental SNPs were shared with *N. sylvestris* and 40 from 56 SNPs with *N. obtusifolia* suggesting its origin from the paternal parent; (iii) none of the parental SNPs were identical to the third cloned sequence (MG545647) and this sequence showed the highest similarity to *N. sylvestris* variant D. This example illustrates the fact that *TERT* sequences from *Nicotiana*s are highly similar (all calculations of similarities show > 90% similarity) and

comparison of query sequences from polyploids to parental sequences of different lengths should be evaluated in detail.

***In silico* and experimental analysis of *TERT* gene copies in *Nicotiana* species.**

To estimate the number of *TERT* gene copies in *N. sylvestris*, *N. tabacum*, *N. tomentosiformis* and *N. benthamiana*, we performed *in silico* analysis. *TERT*-variant specific reads were extracted from raw genomic NGS data from four tested species and verified by blast. Raw reads were mapped to the query (*TERT* exon 9) to elucidate the occurrence of SNPs suggesting the presence of more *TERT* variants. Quantification of the number of mapped reads corresponding to respective *TERT* variants revealed the number and ratio of *TERT* variants (**Table 2**).

Number and ratio of *TERT*_C and *TERT*_D variants was further investigated experimentally in five *N. sylvestris* accessions by qPCR analysis using genomic DNA template and primer combinations according to [1](**Table 3**). Both qPCR reactions had the same reaction efficiency (**Table S3**). To determine detection limits, we performed additional analyses using half the amount of input gDNA templates for the C variant (C 1/2x) to mimic a 2-fold difference in template levels. Results show our experimental set-up is able to determine gene dosage because the ΔCt value (C-C 1/2x) corresponded ca. 0.5 in all tested accessions, clearly discriminating a quantitative difference between one and two copies in genomic DNA input. Subsequent differences in Ct values for NsTERT_C and NsTERT_D specific primer combinations reflect differences between the number of copies of *TERT*_C and *TERT*_D variants. In the case of *N. sylvestris* accessions, the ΔCt (C-D) value was near zero and thus both variants occurred in the *N. sylvestris* genomic DNA in the same ratio (1:1).

The same qPCR analysis was performed using specific primer combinations for the occurrence of *TERT* variants in *N. nudicaulis* genomic DNA. We evaluated primer combinations specific for amplification of TERT_Cs and TERT_D variants originating from *N. sylvestris*, and of the TERT_O variant from *N. obtusifolia*. In the case of NnudiTERT_D and NnudiTERT_O, the variants were amplified using the forward primer 10exF_Nnudi/O/D in combination with specific reverse primers 10exR_Nnudi/SD and 10exR_Nnudi/O, respectively. Variant-specific amplification was tested using restriction digestion with *Mse*I enzyme which cuts within NnudiTERT_O but not within NnudiTERT_D region in qPCR products (**Figure 2C**). qPCR with these specific primer combinations and *N. nudicaulis* genomic DNA as a template confirmed a ratio of 1:1:1 for NnudiTERT_Cs: NnudiTERT_D: NnudiTERT_O variants.

Possible evolutionary scenario and origin of subsequent multiple *TERT* loci in *N. sylvestris*

There is no information about a species-specific WGD event or an additional genome donor in *N. sylvestris* but an increase in transposable elements and repeats was reported [2]. We presume therefore that the ancestral *TERT*_D locus (including *TERT*_12exD and *MtATPO*) originated as a result of gene/segment duplication of the *TERT*_C (plus *MtATPO*) locus or *vice versa*. Both loci were pseudogenized, *TERT*_C within the *MtATPO* region and *TERT*_D within the *TERT* region, and later the *TERT*_D locus was split and translocated by Ogre/SD1-I. Activation of transposable elements was observed as a stress response to genome instability that may be caused by a polyploidization event or environmental stress [2,3]. Crucially, it is difficult to distinguish which of the copies, *TERT*_C or *TERT*_D, was derived from an ancestral copy because the *N. sylvestris* genome assembly is not complete and the mutual positioning of both variants in the genome is unknown.

***In silico* analysis of TERT gene copies in plant genomes.**

In contrast to *P. trichocarpa* and *A. trichopoda*, *M. guttatus* and *V. radiata* shared eudicot-like *TERT* microsynteny (**Figure 5**). Various arrangements of TERT copies are described here and illustrated in **Figure S2**.

Amborella trichopoda (**Figure S3A**) - alignment of nucleotide and corresponding protein sequences of the full-length *TERT* gene (LOC18433477) and a truncated *TERT*-like sequence (LOC18443854) showed low mutual nucleotide and protein sequence similarities (56.7% and 61.9%, respectively;

calculated as a pairwise identity by Geneious), suggesting a possible misassembly. However, both sequences are supported by numerous NGS reads and expression of both *TERT*-like sequences including splicing variants is heavily supported by RNAseq data.

Populus trichocarpa (**Figure S3B**) – The *TERT* gene (Potri.003G022600), located on chromosome 3, represents the full-length gene copy. Predicted *P. trichocarpa TERT* coding gene has a unique exon/intron structure with 10 exons, contrary to the majority of plant *TERT* genes with 12 exons. The *TERT* copy on chromosome 1 (position: 20755289 – 20772968 nt) contains several large indels and stop codons, presumably encoding the *TERT* pseudogene. Based on mRNA data aligned to the genome assembly (Genome ID: 23993), the full-length *TERT* gene is fully transcribed whereas the *TERT* pseudogene transcripts cover only the 650 nt long region corresponding to the 3′end of *TERT*. GEvo alignment of both *TERT*-containing loci showed a large conserved syntelog between 600 kb and 710 kb regions from chromosome 1 and 3, respectively, suggesting that a large-scale segment/genome duplication event had created a novel TERT locus. This scenario illustrates one possible origin of a novel *TERT* locus in ancient *N. sylvestris*.

Mimulus guttatus (**Figure S3C**) – two *TERT* copies were identified using the CoGe platform [4]. According to the analysis of ORF, the *TERT* gene variant (feature ID: 552251894) located at scaffold_2 (position: 15,188,592-15,194,770) represents a functional *TERT* gene copy. The second *TERT* copy (feature ID: 559296275) was placed on the same scaffold (position: 10,362,600-10,366,204) and seems to be non-functional due to the presence of premature stop codons and indels resulting in changes in ORF. Moreover, according to mapped mRNA data, only the 5′end of mRNA of a non-functional *TERT* copy is transcribed (**Figure S3C**). The arrangement of genes neighbouring the functional *TERT* copy is remotely similar to eudicot-like *TERT* synteny (**Figure 4**) but genes in the locus with a non-functional *TERT* are not. However, a limited similarity of both *TERT* loci is obvious because they share two uncharacterized genes adjacent to the *TERT* gene and a 3kb repetitive sequence. This repetitive sequence is highly abundant in the *M. guttatus* genome and interspersed inside and outside the non-functional *TERT* copy in both orientations (illustrated in **Figure S3C**). Thus, we assume these repeats may have mediated or facilitated *TERT* duplication in the *M. guttatus* genome, possibly as an ancient duplication of *TERT* sequences in *N. sylvestris*.

Vigna radiata (**Figure S3D**) – In comparison to previously characterized *TERT* homologs, where one gene copy sustained the original function and another copy was usually an accumulation of mutations or invasions of transposable elements, the additional *TERT*-like sequence in *Vigna radiata* displayed different features. The *TERT* gene copy that shares eudicot-like *TERT* synteny was located on chromosome 5. The second *TERT*-like sequence was located on chromosome 10, within a unique genomic region without any similarity to the *TERT* loci from other plant genomes investigated. This *TERT*-like sequence was annotated as ncRNA in the genome database and it contains two adjacent inverted copies of exon 9 of *TERT*. Although the origin of this *TERT*-like sequence remains unclear, a novel function in *TERT* gene regulation/repression mediated by dsRNA transcribed from the inverted repeats [5] can be expected.

References

1. Jureckova, J.F.; Sykorova, E.; Hafidh, S.; Honys, D.; Fajkus, J.; Fojtova, M. Tissue-specific expression of telomerase reverse transcriptase gene variants in Nicotiana tabacum. *Planta* **2017**, *245*, 549-561, doi:10.1007/s00425-016-2624-1.
2. Renny-Byfield, S.; Chester, M.; Kovarik, A.; Le Comber, S.C.; Grandbastien, M.A.; Deloger, M.; Nichols, R.A.; Macas, J.; Novak, P.; Chase, M.W., et al. Next generation sequencing reveals genome downsizing in allotetraploid Nicotiana tabacum, predominantly through the elimination of paternally derived repetitive DNAs. *Mol Biol Evol* **2011**, *28*, 2843-2854, doi:10.1093/molbev/msr112.
3. Madlung, A.; Tyagi, A.P.; Watson, B.; Jiang, H.; Kagochi, T.; Doerge, R.W.; Martienssen, R.; Comai, L. Genomic changes in synthetic Arabidopsis polyploids. *Plant J* **2005**, *41*, 221-230, doi:10.1111/j.1365-313X.2004.02297.x.

4. Lyons, E.; Freeling, M. How to usefully compare homologous plant genes and chromosomes as DNA sequences. *Plant J* **2008**, *53*, 661-673, doi:10.1111/j.1365-313X.2007.03326.x.
5. Muskens, M.W.; Vissers, A.P.; Mol, J.N.; Kooter, J.M. Role of inverted DNA repeats in transcriptional and post-transcriptional gene silencing. *Plant Mol Biol* **2000**, *43*, 243-260.
6. Sykorova, E.; Fulneckova, J.; Mokros, P.; Fajkus, J.; Fojtova, M.; Peska, V. Three TERT genes in Nicotiana tabacum. *Chromosome Res* **2012**, *20*, 381-394, doi:10.1007/s10577-012-9282-3.

Table S1. List of *Nicotiana* species used in experimental approaches.

Species	Accession name	Source
N. sylvestris	A04750326	Nijmegen Botanical Garden (NL)
N. sylvestris	934750005	Nijmegen Botanical Garden (NL)
N. sylvestris	TW136	USDA (US)
N. sylvestris	ITB626	Tobacco Institute of Bergerac (F)
N. sylvestris	Ducrettet 101-268	Tobacco Institute of Bergerac (F)
N. obtusifolia var. *trigonophyla*	TW143	USDA (US)
N. nesophila	TW87	USDA (US)
N. stocktonii	974750101	Nijmegen Botanical Garden (NL)
N. repanda	TW110	USDA (US)
N. nudicaulis	TW90	USDA (US)
N. paniculata	TW99	USDA (US)
N. undulata	TW145	USDA (US)
N. rustica		Queen Mary University of London (UK)
N. quadrivalis	ITB101-234	Tobacco Institute of Bergerac (F)
N. clevelandii	TW30	USDA (US)
N. wigandoides	ITB630	Tobacco Institute of Bergerac (F)
N. attenuata	92475008	Nijmegen Botanical Garden (NL)
N. arentsii	ITB571	Tobacco Institute of Bergerac (F)
N. alata		Queen Mary University of London (UK)
N. benthamiana		Queen Mary University of London (UK)

Table S2. List of primers used in this study.

PRIMER	SEQUENCE (5´→ 3´)	METHOD
4exF<br>5exR<br>9exF1<br>9exR<br>10exF<br>10exR<br>12exR	CCCTCGAAACAAACACCGTCAG<br>CCTTTTCAAGAAGAAGCACTGAA<br>TTCCAAGCTTGTTAAGCTACGAAGG<br>CAAGAATGCCATGCAATGAGCC<br>CAATGTGCTGCGGTTAGGTAGC<br>AGAAGAACCTAGAAGCCTGTTCC<br>AATCCGAGCCATTCGATTTCACC	Primers from [6] used in screening experiments
9exF3<br>9exR_Nsyl/C<br>9exR_Nsyl/D	GTGCTGGATGATCTCAATTTGGAG<br>GGAAGATAAGACCTAGCATTGGCG<br>GAAGACAAGACCTGGCATTGACG	Primers used in variant specific TERT RTqPCR in *N.sylvestris* from [1]
9exF3<br>9exR_Nrust/P<br>9exR_Nrust/U	GTGCTGGATGATCTCAATTTGGAG<br>CAGTGGGAAGAGAAGACCCA<br>CAGTGGGAAGACAAGACCTG	Primers used in variant specific TERT RT-qPCR in *N.rustica*
9exR_Nrepa/S<br>9exR_Nrepa/O<br>9exF2	TTGAGATCATCCAGCACACTCA<br>ATTGAGATCATCCAGCACAGTAG<br>CTTTCTTATCACTCCTGCAGAGTG	Primers used in variant specific TERT RT-qPCR in *N.repanda*
10exF_Nnudi/C<br>10exF_Nnudi/O/D<br>10exR_Nnudi/SC<br>10exR_Nnudi/SD<br>10exR_Nnudi/O	CCTTCGGAGGAATGTTTTCTC<br>TCCTTCAGAGGAATGTTTTCTT<br>CCACATACGATCTATCTGATCTT<br>AACCACATACAATCTATCCGATAA<br>CTCAGATCTGATAGATTATCTGAA	Primers used in variant specific TERT RT-qPCR and qPCR in *N.nudicaulis*

Table S3. Primer combinations used in screening, qPCR and RT-qPCR experiments, and sequences submitted to Genbank

	Species	Primer combinations/no. of detected *TERT* variants*	Genbank accessions*
Screening	*N. alata*	4exF x 5exR/1	MG242421
	N.obtusifolia	9exF1 x 9exR/1;10exFx12exR/1	MG242404,MG242417
	N. undulata	9exF1 x 9exR/1	MG242412
	N. wigandiodes	9exF1 x 9exR/1<br>4exF x 5exR/1	MG242420<br>MG242424
	N. paniculata	9exF1 x 9exR/1	MG242411
	N. attenuata	4exF x 5exR/1	MG242425
	N.benthamiana	9exF1 x 9exR/1	
	N.repanda	4exF x 5exR/1; 9exF1 x 9exR/2;10exFx12exR/2	MG242415, MG242416<br>MG242402, MG242403
	N.stocktonii	4exF x 5exR/1; 9exF1 x 9exR/2;	MG242407, MG242408
	N.nesophila	4exF x 5exR/1; 9exF1 x 9exR/2;	MG242405, MG242406
	N.nudicaulis	4exF x 5exR/1; 9exF1 x 9exR/1; 9exF1 x 9exR_Nrepa/O/0; 10exFx10exR/2; 10exFx12exR/3	MG242409, MG242410, MG545647
	N.clevelandii	4exF x 5exR/1;	MG242422
	N.quadrivalvis	4exF x 5exR/1; 9exF1 x 9exR/1; 10exFx10exR/1	MG242423
	N.arentsii	4exF x 5exR/1; 9exF1 x 9exR/2	MG242418, MG242419
	N.rustica	9exF1 x 9exR/2	MG242413, MG242414
RT-qPCR and qPCR analyses	**Species**	**primer combinations**	**PCR efficiency**
	N.sylvestris	9exF3 x 9exR_Nsyl/C (for amplification of C variant in *N.sylvestris*)	1.00
		9exF3 x 9exR_Nsyl/D (for amplification of D variant in *N.sylvestris*)	1.00
	N.repanda	9exF2 x 9exR_Nrepa/S (for amplification of C variant from *N.sylvestris*)	1.03
		9exF2 x 9exR_Nrepa/O (for amplification of variant from *N.obtusifolia*)	1.08
	N.rustica	9exF3 x 9exR_Nrust/P (for amplification of variant from *N.paniculata*)	1.07
		9exF3 x 9exR_Nrust/U (for amplification of variant from *N.undulata*)	1.08
	N.nudicaulis	10exF_Nnudi/C x 10exR_Nnudi/SC (for amplification of C variant from *N.sylvestris*)	0.95
		10exF_Nnudi/O/D x 10exR_Nnudi/SD (for amplification of D variant from *N.sylvestris*)	1.02
		10exF_Nnudi/O/D x 10exR_Nnudi/O (for amplification of variant from *N.obtusifolia*)	0.93

* primer combinations corresponding to sequences of representative clones submitted to Genbank

Table S4. Origin of TERT gene variants in polyploid *Nicotiana* species determined by number of SNPs homologous to representative progenitor diploids

Allopolyploids	Genebank accessions	SNP homology (X/Y), (%)[1]				analysed region
		maternal parent		paternal parent		
**SUAVEOLENTES**		*N.alata*	*N.noctiflora*	*N.syl.C var.*	*N.syl. D var.*	
N.benthamiana	NbS000104 27g0116.1	4/19	n.a.	**14/19**	n.a.	exon 4 to 5
		n.a.	8/30	**20/30**	93.4 %	exons 10,11,12
**REPANDAE**		*N.syl. C var.*	*N.syl. D var.*	*N.obtusifolia*		
N.repanda	MG242402	0/18	91.6 %	**16/18**		exon 9
	MG242403	**16/18**	92.4 %	2/18		exon 9
N.stocktonii	MG242407	0/18	91.7 %	**17/18**		exon 9
	MG242408	**16/18**	93.1 %	0/18		exon 9
N.nesophila	MG242405	0/18	91.6 %	**15/18**		exon 9
	MG242406	**18/18**	92.9 %	0/18		exon 9
N.nudicaulis	MG242409	**42/56**	94.3 %	2/56		exon 10 to 12
	MG545647	0/56	**94.8 %**	0/56		exon 10 to 12
	MG242410	7/56	93.3 %	**40/56**		exon 10 to 12
**POLYDICLIAE**		*N.obtusifolia*		*N.attenuata*		
N.clevelandii	MG242422	0/29		**29/29**		exon 4 to 5
	var1[2]	*6/32*		**23/32**		*exon 9*[2]
	var2[2]	**21/32**		**5/32**		*exon 9*[2]
N.quadrivalvis	MG242423	0/29		**27/29**		exon 4 to 5
**ARENTSII**		*N.undulata*		*N.wigandiodes*		
N.arentsii	MG242418	**7/8**		0/8		exon 9
	MG242419	0/8		**7/8**		exon 9
**RUSTICA**		*N.paniculata*		*N.undulata*		
N.rustica	MG242413	**13/13**		0/13		exon 9
	MG242414	1/13		**12/13**		exon 9

[1](X/Y) - number of SNP homologous to parental TERT (X) related to the number of differential SNPs found between parental TERTs (Y), see above and Figure S1 for description. The variant D is present in *N. sylvestris* progenitor diploid only thus SNPs cannot be analysed; sequence similarity in (%) is shown instead.

[2] sequences from *N. clevelandii* and its progenitor diploids are in Appendix A1

Table S5. List of plant species, genome accessions and *TERT* loci analysed *in silico* using Synfind and GEvo.

Species	Genome ID	TERT (Feature name)
Solanum pennellii	28941 (v2.0)	Sopen01g034170.1.10
Solanum lycopersicum	35173 (v3.10)	Solyc01g087095.1.1.10
Capsicum annuum	22828 (v2.0)	Capang01g002261
Nicotiana tomentosiformis	12239 (v1)	LOC104105435
Nicotiana benthamiana	20448 (v0.4.4)	NbS00010427g0116.1
Mimulus luteus	22656 (v1.1)	FID:566839053, FID:548989864
Mimulus guttatus	22665 (v2)	FID:552251894, FID:559296275
Sesamum indicum	26082 (v1.0)	cds1981
Lactuca sativa	28333 (v8)	Lsat_1_v5_gn_4_31580.1
Actinidia chinensis	20457 (v1.0)	Achn298511-TA
Vitis vinifera	19990 (v12x)	PAC:17838116
Fragaria vesca	3314 (v1)	LOC101312102
Prunus persica	34844 (v2.0)	Prupe.2G012600.4.v2.1
Glycine max	5(v2.0)	LOC100790649, LOC100776816
Phaseolus vulgaris	19521 (v1.0)	PAC:27150484
Cicer arietinum	32935 (v1.0)	cds14997
Vigna radiata	NC_028355	LOC106760534, LOC106775712
Cucumis sativus	19927 (v2.0)	Csa3M697400.1
Populus trichocarpa	8154(v2.0)	POPTR_0003s01700, POPTR_0001s21470.1
Manihot esculenta	10538 (v4.1)	cassava4.1_022790m
Ricinus communis	7743 (v2.0)	RCOM_0816800
Eucalyptus grandis	2181 (v1)	LOC104420141
Citrus sinensis	10702 (v1.0)	LOC102625932
Tarenaya hassleriana	34654(v5.1)	Th2v09108
Arabidopsis thaliana	16911(v10.02)	AT5G16850.1
Camelina sativa	14087 (v1)	LOC104705979, LOC104735722, LOC104769972
Brasicca oleracea	10901	LOC106317925
Brassica napus	203 (v2)	LOC106371945, LOC106387699
Brassica rapa	24668(v1.5)	Bra008588
Theobroma cacao	35189(v1)	TCM_044678
Gossypium hirsutum	10704	LOC107927522
Nelumbo nucifera	16884 (v2.0)	NNU_007864
Oryza sativa	9218 (v5)	Os12g0293100
Brachypodium distachinon	698	LOC100830375
Setaria italica	12240(v2.1)	Si021730m.g
Sorghum bicolor	108 (v3)	Sb01g034910
Zea mays	12(v4)	GeneID:732781
Ananas comosus	25735 (v6)	Aco021544.1
Musa balbisiana	22639 (v1)	ITC1587_Bchr8_P21968
Elaeis guineensis	2669	LOC105032643
Asparagus officinalis	33908(v2)	AsparagusV1_06.1405
Amborella trichopoda	12031(v1.0)	LOC18433477; LOC18443854
Selaginella moellendorffii	1636 (v1)	SELMODRAFT_171959
Physcomitrella patens	7695 (v1)	Pp3c1_42700V1.1
Chlamydomonas reinhardtii	16710 (v1)	Cre04.g213652.t1.1.v5.5
Chlorella variabilis	14389 (v1)	CHLNCDRAFT_32983
Micromonas pusilla	16401 (v1)	EEH55489.1
Ostreococcus tauri	18596 (v1)	Ot04g03700
Volvox carteri	26074 (v2)	Vocar20001997m.2.0

Figure S1. Illustration of SNP analysis and determination of *TERT* sequence origin. SNPs between representatives of parental sequences were identified. The identity and number of SNPs in polyploids (short region of representative clones from *N. repanda* is shown) identical/homologous with parental SNPs (*N. sylvestris, N. obtusifolia*) were determined using multiple alignment of *TERT* sequences. Thus subsets of all SNPs were individually evaluated because clone/variant-specific SNPs from polyploids were not appraised.

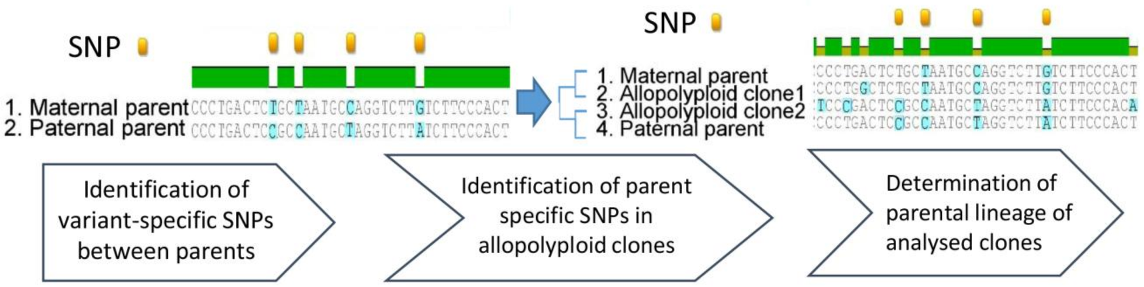

Figure S2. Possible scenario for maintaining multiple *TERT* copies in polyploid genomes. Reconstruction of a possible scenario (derived from current genomes) starts with the situation in *Amborella*, i.e. the *TERT* gene is located in a specific locus (violet/pink) and novel loci that have accommodated the *TERT* gene in current species are already present in the *Amborella* ancestral genome. After *TERT* gene translocation into a novel eudicot-like locus (yellow/green), supported by *Nelumbo* data, the original locus is fragmented. Multiplication of *TERT* gene copies could be achieved by various events – whole genome/segment/gene duplications. Our results from *Nicotiana* and other genomes (examples listed in schemas) could illustrate subsequent processing of *TERT* copies including their fractionation, pseudogenization, elimination or possible neofunctionalization. Copies of genomic loci with the original synteny remain present after *TERT* gene elimination, e.g., in *Actinidia chinensis*, where it is difficult to distinguish which of the ohnologous loci (ohnologs = paralogs derived by WGD) have lost the *TERT* gene copy. Note there is also a slight possibility that the current *Amborella* locus with *TERT* gene may reflect a translocation of *TERT* from an unknown ancestral locus into the current *Amborella*-specific locus that is not present in genomes investigated, however; these various options are not discussed.

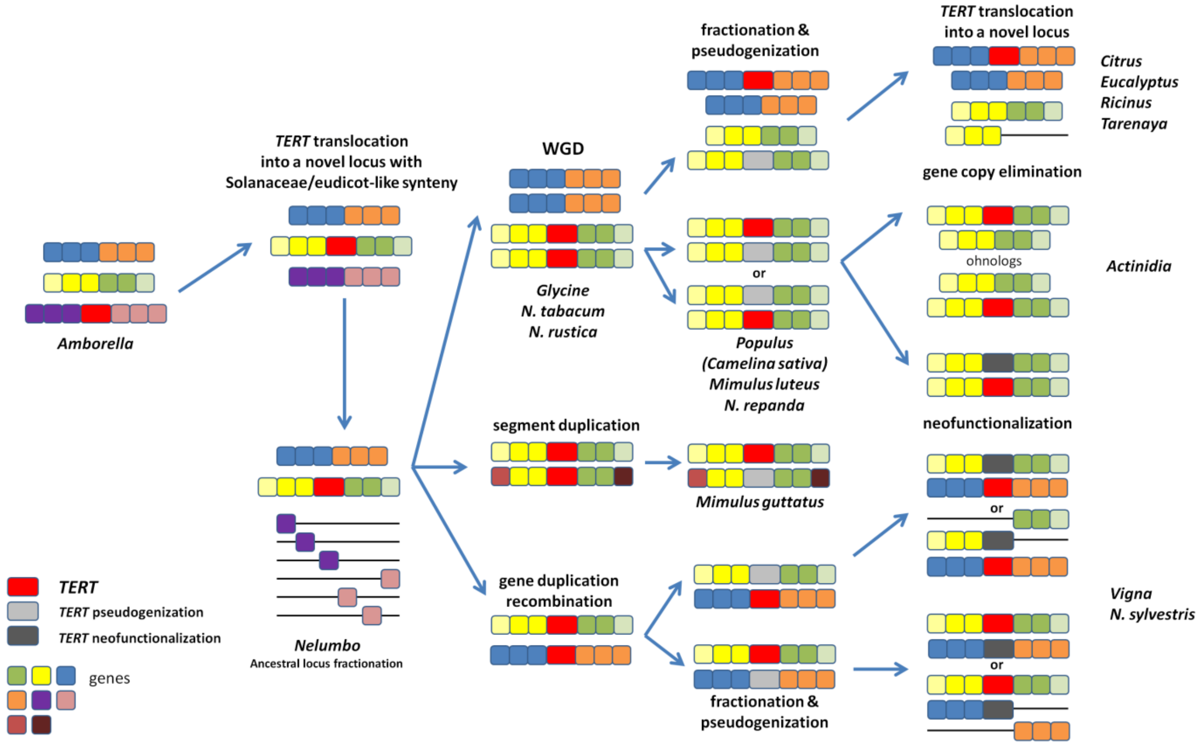

Figure S3. Examples of additional copies of *TERT*-like sequences identified in plant genomes. (A) The full-length *TERT* and the truncated *TERT* sequence were identified in *Amborella trichopoda*. The predicted exon/intron structure of both *TERT*-like sequences and alignment of predicted cDNA sequences are shown. The truncated variant covers the N-terminal part of the TERT protein including the telomerase specific T2 motif (position depicted). (B) GEvo comparison of the *TERT* loci from chromosome 1 and 3 of *Populu*s *trichocarpa* illustrates the duplication of the *TERT* region and pseudogenization of the *TERT* copy on chromosome 1. (C) Dotplot of two 40 kb long genomic loci with *TERT*-like sequences (in blue) from *Mimulus gutatus* show hits for neighbouring genes (in black boxes) and amplification of repetitive sequence (in red boxes) interspersed inside and outside of a non-functional *TERT* copy. (D) An additional *TERT*-like sequence (in LOC106775712) in the *Vigna radiata* genome with similarity to the functional *TERT* gene (in LOC10676034) is annotated as ncRNA. Dotpot visualisation identified two inverted copies corresponding to the exon 9 region (boxed) and two hits with similarity to intron 7 and exon 8 regions of *TERT* within the ncRNA sequence.

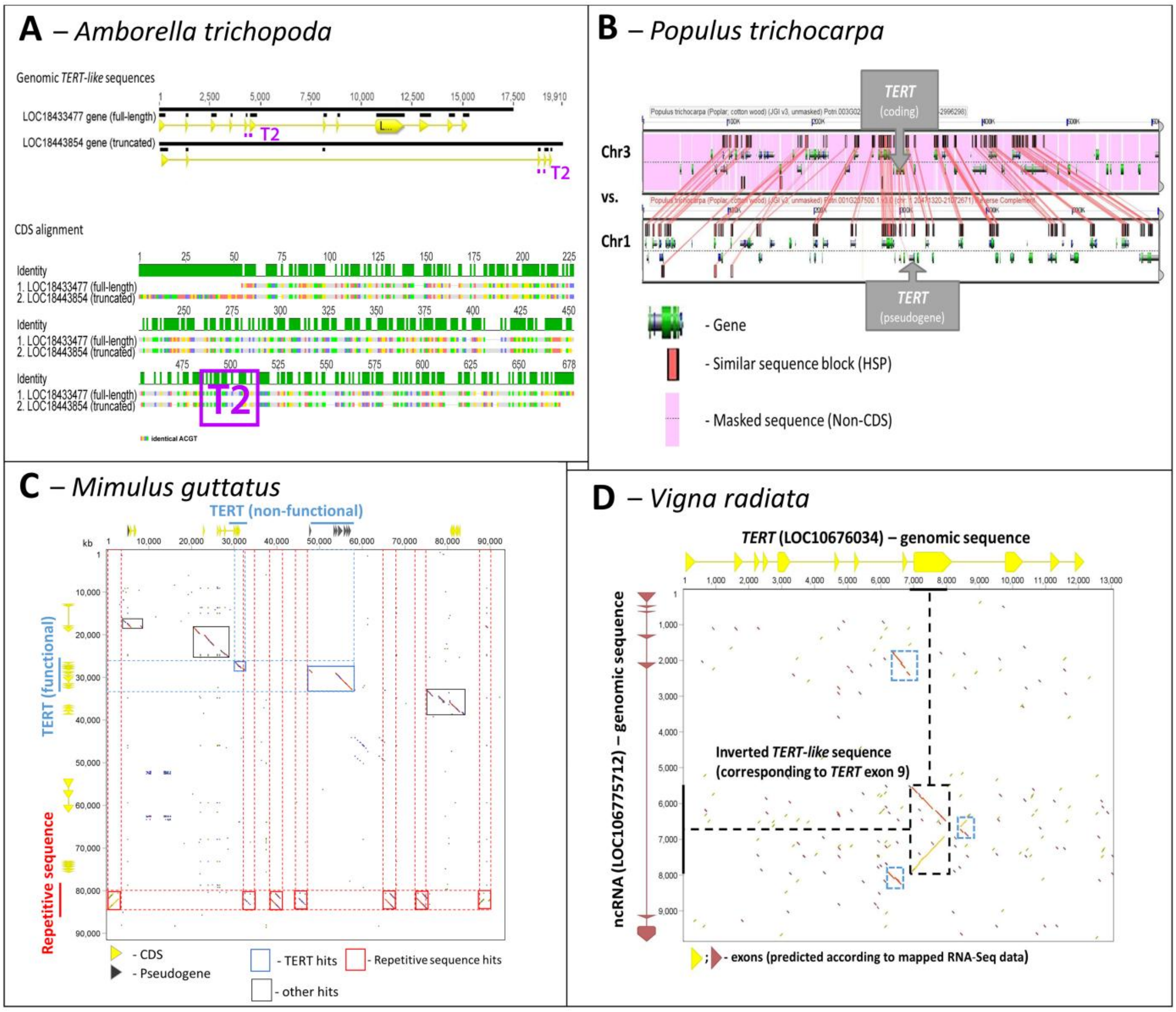

Supplement: Supplementary file 1 [file ijms-22-01783-s001.zip › Supplementary_material_final.pdf]
